# Triazine Herbicide and NPK Fertilizer Exposure: Accumulation of Heavy Metals and Rare Earth Elements, Effects on Cuticle Melanization, and Immunocompetence in the Model Species *Tenebrio molitor*

**DOI:** 10.3390/toxics11060499

**Published:** 2023-06-01

**Authors:** Attilio Naccarato, Maria Luigia Vommaro, Domenico Amico, Francesca Sprovieri, Nicola Pirrone, Antonio Tagarelli, Anita Giglio

**Affiliations:** 1 Department of Chemistry and Chemical Technologies, University of Calabria,87036 Rende, Italy; attilio.naccarato@unical.it (A.N.); antonio.tagarelli@unical.it (A.T.); 2 Department of Biology, Ecology and Earth Science, University of Calabria, 87036 Rende, Italy; 3 CNR-Institute of Atmospheric Pollution Research, 87036 Rende, Italy; domenico.amico@iia.cnr.it (D.A.); f.sprovieri@iia.cnr.it (F.S.); pirrone@iia.cnr.it (N.P.)

**Keywords:** agroecosystem, cuticular darkness, chemometric analysis, immunocompetence, mealworm beetle, multielement profile, sexual maturation, risk assessment

## Abstract

The increasing use of agrochemicals, including fertilizers and herbicides, has led to worrying metal contamination of soils and waters and raises serious questions about the effects of their transfer to different levels of the trophic web. Accumulation and biomagnification of essential (K, Na, Mg, Zn, Ca), nonessential (Sr, Hg, Rb, Ba, Se, Cd, Cr, Pb, As), and rare earth elements (REEs) were investigated in newly emerged adults of *Tenebrio molitor* exposed to field-admitted concentrations of a metribuzin-based herbicide and an NPK blend fertilizer. Chemical analyses were performed using inductively coupled plasma tandem mass spectrometry (ICP-MS/MS) supported by unsupervised pattern recognition techniques. Physiological parameters such as cuticle melanization, cellular (circulating hemocytes), and humoral (phenoloxidase enzyme activity) immune responses and mass loss were tested as exposure markers in both sexes. The results showed that NPK fertilizer application is the main cause of REE accumulation in beetles over time, besides toxic elements (Sr, Hg, Cr, Rb, Ba, Ni, Al, V, U) also present in the herbicide-treated beetles. The biomagnification of Cu and Zn suggested a high potential for food web transfer in agroecosystems. Gender differences in element concentrations suggested that males and females differ in element uptake and excretion. Differences in phenotypic traits show that exposure affects metabolic pathways involving sequestration and detoxification during the transition phase from immature-to-mature beetles, triggering a redistribution of resources between sexual maturation and immune responses. Our findings highlight the importance of setting limits for metals and REEs in herbicides and fertilizers to avoid adverse effects on species that provide ecosystem services and contribute to soil health in agroecosystems.

## 1. Introduction

The growing global demand for food to feed humans and livestock has led to an increase in agricultural activities and the application of annually increasing amounts of fertilizers [1] to supply macro- and micronutrients to soil, as well as plant protection products including herbicides.

Fertilizers are broadly classified into inorganic fertilizers, which include nitrogenous, phosphate, potassium, and complex fertilizers, and organic fertilizers such as farmyard manure, bone meal, compost, and green manures. NPK blends are the most commonly used mineral fertilizers in agriculture [2,3]. They contain the three basic plant nutrients, i.e., nitrogen (N), phosphorus (P), and potassium (K) in concentrations that vary with soil and crop type, and are often enriched with Ca, Mg, and micronutrients such as Fe, Mn, Zn, B, Mo, and Cu together act on plant metabolism to improve crop growth and production [4]. In addition, commercial fertilizers have been shown to contain trace metals such as Cd, As, Cr, Zn, Ni, and Pb in varying concentrations depending on the geographic origin of phosphate rocks [5,6] or livestock manure and sewage sludges [7]. The first concern that arises in the context of agricultural pollution control is the alteration of the physical and chemical properties and fertility of soils, leading to acidification and nitrification, and the eutrophication of water bodies due to fertilizers runoff, resulting from their long-term application [8,9]. This is also a consequence of the fact that the market of fertilizers is not subject to strict regulation and has been partially harmonized by European regulation [10].

Herbicides are chemicals used as pre- and post-emergence treatments to selectively inhibit weeds by acting on photosynthesis, pigments, cell membrane, cell growth, lipids, and amino acid synthesis [11]. Currently, they account for about 50% of the annual amount of chemicals used in agricultural soils to protect crops [12,13]. However, herbicides have been found to cause side effects on non-target species at all levels of the trophic web [14,15,16,17,18] and the alteration of communities in terrestrial [19,20] and aquatic ecosystems [21]. In agricultural systems, herbicides applied to soil can affect soil-dwelling arthropods [22], exposed by direct spraying or drift, including insects involved in pest control [23] and pollination [24,25], and disrupt basic symbiotic associations [26,27,28]. Recent studies have also found that the ecological risk of commercial herbicide formulations depends primarily on adjuvants added to enhance the absorption and stability of the active ingredient [29] as well as metalloids and metals added due to impurities during the manufacturing processes or as nanoparticles to accelerate cell penetration, often resulting in concentrations above permitted levels [30,31]. Triazines are chemicals used for pre- and post-emergence weed control. They are taken up through the roots and transported upward within the plant to the leaves and cause the inhibition of photosynthesis by blocking electron transport binding a protein subunit of photosystem II [32]. These include metribuzin [4-amino-6 t-butyl-3-methylthio)-1,2,4 triazine-5(4H) one], which is used worldwide on potatoes, soybeans, sugarcane, wheat, and other crops. Its persistence in the environment is short to moderate depending on soil pH [32]. This herbicide has been shown to have adverse effects on terrestrial and aquatic nontarget organisms. In earthworms, it has been reported to interfere with growth, survival [33], and nervous and immune systems [34]. Effects on populations have been noted in soil-dwelling mites [35] and curculionid alfalfa weevils [36]. In addition, genotoxic effects have been tested in *Drosophila melanogaster* [37], ground beetles [38], and bumblebees as pollinators [39]. Sublethal effects on hematological, biochemical, and histopathological parameters occurred in fish, [40,41], crustaceans [42], mammals [43], and humans [44].

Current data indicate that intensive and continuous applications of fertilizers and agrochemicals (i.e., insecticides, herbicides, fungicides), used to ensure successful crop production, enrich soil with potentially harmful levels of metals such as As, Cd, Pb, and Hg [5,45,46,47], which are on the World Health Organization [48] list of priority substances of environmental and public health concern due to their toxicity, and essential metals, e.g., Cu, Co, Fe, Se, and Zn, which are required for biochemical and physiological processes and are toxic above certain concentrations [49,50]. Recent studies have shown that metal concentrations exceed the permissible threshold in agricultural areas of different countries with different soil types and management practices [47,51,52,53,54,55] in Europe, this occurs on 173 million hectares of agricultural land [51,56]. Such accumulation in the soil leads to the uptake and translocation of metals at different levels of the trophic web in agroecosystems [57,58,59,60], with a negative impact on biodiversity and human health [55,61]. The importance of this framework and the urgency of timely action to monitor and preserve agricultural soils are also reflected in the United Nations Sustainable Development Goals (SDGs), which directly and indirectly address land and soil and call for strong efforts to reduce the impact of agriculture on the environment and communities while providing high-quality, nutritious food for a local and broad consumer base [62]. The new EU Fertilizing Products Regulation (EU) 2019/1009 [10] introduced limits for some elements such as Cd, Cr VI, Hg, Ni, Pb, As, B, Co, Cu, Fe, Mn, Mo, and Zn in organic and inorganic fertilizers. However, for commercial products such as herbicides and insecticides, there is no indication of approved metal contaminants, although the active ingredient and commercial formulations must be approved for distribution, sale, and use [63,64]. Moreover, Regulation (EU) 2019/1009 lacks any references to the use of rare earth elements (REEs) and the permissible limits in both organic and chemical fertilizers. Fertilizers are enriched with REEs to promote seed germination and improve crop growth and production [65,66,67], further increasing concentrations of these elements in soil [68]. Despite the high demand for these substances for various applications, there are still significant gaps in the understanding of their negative effects on human health and biodiversity [67,68,69,70,71,72,73].

Knowledge of the transfer of heavy metals and REEs through trophic networks is limited. Invertebrates, especially insects, play a key role in agroecosystems, providing services such as pollination, pest and weed control, and nutrient recycling [74]. However, these organisms are obviously exposed to chemical treatments in crops that can directly or indirectly interfere with the multiple interspecific relationships they establish within agroecosystem communities [75]. Beetles have proven to be a reliable model in ecotoxicology because they are highly sensitive to environmental perturbations. Furthermore, they are representative of environmental diversity and supply a high degree of standardization [76], such as *Tenebrio molitor* Linnaeus, 1758 (Coleoptera, Tenebrionidae), a pest of stored grain, used as a model in experimental science, due to its well-characterized ecology and physiology [77,78].

In the present study, we used inductively coupled plasma tandem mass spectrometry (ICP-MS/MS) to determine the concentration of the multielement profile in a metribuzin-based herbicide and an NPK-blended fertilizer commercial formulation. Then, to measure the accumulation of trace elements in the beetles and their biomagnification, newly emerged adults of mealworm beetle *T. molitor* were exposed in the laboratory to concentrations of these herbicide and fertilizer formulations admitted in the field. Finally, we tested immune parameters, such as the change in the enzyme activities of the plasmatic phenoloxidase (PO) and the total number of circulating hemocytes, body mass, and cuticle darkness as markers of sublethal effects [79]. The markers chosen to monitor the response to exposure in a time window critical for cuticle browning and sexual maturation are involved in vital functions and have been shown to be highly plastic in their regulation [80]. Hemocytes and antimicrobial peptides including PO enzyme cascades are cellular and humoral effectors involved in counteracting pathogens that break the barrier of the cuticle, epidermis, or gut epithelium [78,81]. The melanization of pathogens and cuticle browning in insects both depend on tyrosine metabolic pathways [82,83]. The cuticle is the first line of defense and is involved in several basic functions, including support, protection from dehydration and ultraviolet light, sexual signaling, and mimicry [84,85]. Cuticle characteristics could affect the integrity of these processes [86,87], and thus, directly and indirectly, influence the fitness of individuals. In the mealworm beetle, the darkening process is representative of the degree of sclerotization, cross-linking, and melanization of the cuticular layers [88] and is closely related to pathogen resistance [89,90].

The aim of our study was to investigate the sex-related accumulation and biomagnification rate of heavy metals and REEs following exposure to herbicides and fertilizers in *T. molitor* to assess the physiological effects over time. We hypothesized that the exposure of non-target insects to trace elements during the highly sensitive transitional phase from newly emerged to sexually mature adults may impair life traits such as sexual maturation, immune response, and detoxification affecting individual fitness. Better understanding elements’ accumulation as a result of chemical treatments in the field could help preserve and restore insect communities, commonly exposed to agrochemical treatments, that are critical to ecosystem functioning and the sustainable management of agricultural systems for successful biodiversity conservation.

## 2. Materials and Methods

### 2.1. Animal Rearing

Specimens of *T. molitor* were obtained from a laboratory stock population housed in the Laboratory of Morphofunctional Entomology, Department of Biology, Ecology and Earth Science (University of Calabria). The beetles were reared at 60% relative humidity with a natural photoperiod and room temperature (23 ± 2 °C). They were fed ad libitum with wheat flour and fruits. They do not require ethical permission to use.

### 2.2. Herbicide and Fertilizer

Commercial formulations of a metribuzin-based herbicide (Feinzin 70 DS, Adama Bergamo, Italia Co. Ltd., Grassobbio, Italy, a.i. metribuzin 70%; **MTB**) and a fertilizer (ATB plus Timagreen Timac Agro, Italia Ltd.,(Cremona, Italy) ingredients in percent NPK:10-5-12, CaO-MgO-SO_3_: 8-2-24, 0.1 B and 7.5 C; **TG**) were used in this study. The formulations were stored at room temperature in their original packaging. Stock solutions were freshly prepared and diluted with distilled water at room temperature. The recommended field doses of both formulations, indicated for cereal and vegetable crops, were tested as indicated in the data sheets, i.e., 0.25 kg ha^−1^ of **MTB** and 4 × 10^2^ kg ha^−1^ of **TG**. The wheat bran used for feeding beetles of the control, MTB, and TG groups was purchased from a mill in the province of Catanzaro (Calabria, Italy).

### 2.3. Experimental Setup and Treatments

Pupae of *T. molitor* were separated by sex, and the newly emerged adults were housed individually in cups (30 mL in volume) with a perforated lid to allow air exchange and provided with 0.5 g of bran which had been previously UV-sterilized to prevent mold contamination.

**Figure 1:**
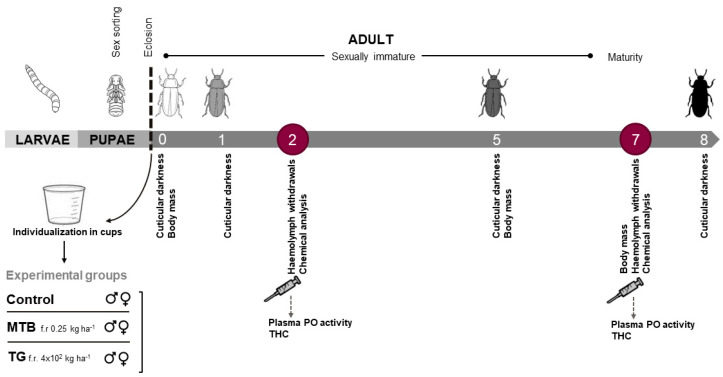
Experimental setup. *T. molitor* pupae were divided by sex, and newly emerged adult beetles were housed in cups and exposed to metribuzin-based herbicide (**MTB**) and NPK-based fertilizer (**TG**) at the recommended field rate for 8 days. A control group was run in parallel. The beetles were assessed during sexual maturation for: physiological and immunological markers—body condition (N = 30), cuticular darkness (N = 30), plasma phenoloxidase (PO) enzyme activity (N = 15), and total hemocyte count (THC; N = 11)—and chemical analysis (N = 20) to assess bioaccumulation and biomagnification.

The exposure of beetles to herbicide and fertilizer was established in a completely randomized design for males and females by adding 150 µL of the recommended field dose solutions (see Section 2.2) administered on filter paper in the respective groups (MTB: 2.25 × 10^−2^ mg in distilled water per cup; TG: 36 mg in distilled water per cup; calculated proportionally on a filter paper surface of 9 cm^2^), while it was soaked with the vehicle only (150 µL of distilled water) in the control group (**C**). The wet filter paper was the only source of moisture for the insects and was kept moist by adding 150 μm of sterile phosphate-buffered saline (PBS, 10 mM; Sigma-Aldrich, Milan, Italy) to all groups on alternate days. The first set of beetles exposed to MTB (20 males and 20 females), TG (20 males and 20 females), and the control group (C, 40 males and 40 females) was used to evaluate the accumulation of the trace element according to the chemical analysis protocol below. The second experimental group was formed to measure the relationship among treatment exposure, sex, and age in terms of immunological and physiological (body mass, cuticle darkness) markers. Beetles were exposed to each treatment with MTB and TG for a total of 8 days (Figure 1). The duration of the exposure was chosen to follow the progression through the sexual maturation interval. To ensure repeatability, each experiment was performed in duplicate, and beetles were exposed individually as single biological replicates.

### 2.4. Chemical Analyses

Chemical analyses of beetles from the treated (MTB and TG) and control (C) groups at 2 and 7 days after the initial exposure included a drying step in an oven at 105 °C for 24 h and the microwave-assisted acid digestion of the samples using an Ethos-Up microwave extraction system (MW) (Milestone, Germany). Samples were prepared as described by Naccarato et al. [58], integrating some modifications. Briefly, a mixture of nitric acid and hydrogen peroxide was used to digest each beetle (approximately 40 mg dry weight each), with the latter component of the mixture added to increase the oxidizing power of the acid, to promote the rapid and complete dissolution of samples [91,92]. In the current study, digestion was performed using vessel-inside-vessel technology (Milestone, Germany), where the digestion is performed in smaller secondary vessels that are placed inside the larger traditional vessel. This setup reduces the amount of chemicals used for digestion, resulting in economic and environmental benefits. The small amount of digestion mixture also allows for an improvement in detection limits by lowering the dilution factor. This is particularly useful for the analysis of trace elements in small amounts of matrix. The use of smaller vessels allows the throughput of each MW run to triple, thereby reducing the environmental and economic cost of preparing each sample. The procedure used in our study involved the use of 10 mL of H_2_O and 1 mL of H_2_O_2_ in the larger vessel in which the temperature control was performed, while in the digestion vessels, each insect was mineralized with a mixture composed of 1.5 mL of HNO_3_ and 0.5 mL of H_2_O_2_. The same procedure was used to digest the reference samples of wheat bran that was used to feed beetles during the experiment, which were digested (100 mg each) in the control and treated (TG and MTB) groups.

The concentrations of 47 elements (Li, Be, Na, Mg, Al, K, Ca, Sc, V, Cr, Mn, Fe, Co, Ni, Zn, Ga, As, Se, Rb, Sr, Y, Ag, Cd, In, Cs, Ba, La, Ce, Pr, Nd, Pm, Sm, Eu, Gd, Tb, Dy, Ho, Er, Tm, Yb, Lu, Hg, Tl, Pb, Bi, Th, U) were determined by inductively coupled plasma–mass spectrometry using triple quadrupole technology (ICP-MS/MS, iCAP TQe, Thermo-Fisher) in treated and control insects, wheat bran, herbicide and fertilizer used for the exposure treatments. The triple quadrupole system enhances the already-high sensitivity of ICP systems and reduces the interferences that limit the performance of conventional single analyzer instruments [93,94,95]. The instrument performance was verified according to the manufacturer’s instructions prior to performing the analyses. Compliance with a satisfactory level of accuracy (within ±20%) and precision (CV% < 5%) of the quantitative determination was regularly evaluated by the analysis of procedural blanks spiked with the elements of interest at three concentration levels within the linear dynamic range covered.

Sample digestion was carried out using nitric acid 65%, Suprapur^®^ for trace analysis (Supelco), and hydrogen peroxide 30% Suprapur^®^ for trace analysis (Supelco). Quantitative analysis was carried out using calibration curves built by diluting multielement solutions (Al, As, Ba, Be, Bi, Cd, Ca, Cs, Cr, Co, Cu, Ga, In, Fe, Pb, Li, Mg, Mn, Ni, K, Rb, Se, Ag, Na, Sr, Tl, U, V, Zn (10 mg/L, VWR); Ce, Dy, Er, Eu, Gd, Ho, La, Lu, Nd, Pr, Sc, Sm, Tb, Th, Tm, Y, and Yb (10 mg/L, Perkin Elmer); and single element solution of Hg (1000 mg/L, Merck)). Each batch of analysis was composed of the standards used for calibration, the procedural blanks, and the samples. The investigated elements were for the most part analyzed in kinetic energy discrimination mode (KED-mode) operating in the helium gas collision cell (99.999% grade). In contrast, Sc, V, Cr, As, Se, Sr, Y, Ce, Pr, Nd, Sm, Gd, Tb, Dy, Ho, Er, Tm, and Lu were quantified in triple quadrupole mode (TQ-mode) using oxygen (99.999% grade) as the reaction gas to reduce the effects of isobaric interferences and improve the sensitivity.

### 2.5. Body Conditions and Cuticular Darkness

The beetles (30 replicates for each experimental condition) were weighed to determine body mass (Ohaus balance, accuracy of 10^−^⁵ g). The measurement was recorded at three different time points in males and females exposed to MTB and TG and in the C group to evaluate the change in body mass during the experiment. Body mass was recorded in newly emerged adults (0 days) before exposure and 5 and 7 days after eclosion, when beetles begin to feed (Figure 1). Relative mass loss was calculated as in [96]. Briefly, the difference between the initial mass and the final mass divided by the initial mass of a given individual was calculated between 0 and 5 days, 5 and 7 days, and the entire experimental period (0 and 7 days).

To track cuticle darkening over the 8 days following eclosion, elytra color was analyzed according to the protocol described by Thompson et al. [97]. Briefly, beetles from each experimental group (MTB, TG, and C; Figure 1; 30 replicates for each experimental condition, separately for males and females) were observed under a stereomicroscope (Zeiss Stemi SV11) and photographed with a camera (Optica C-P8) at 0, 1, 5, and 8 days after eclosion. The difference between the first two time points has been reported in the literature [98] as the most severe variation, while the intermediate points (5 days) were recorded to track the browning until the cuticle reached maximum darkness (day 8). One digital image of each beetle was taken at each time point, under diffuse indirect illumination with a constant-intensity light source. The digitized images were converted to 8-bit files using Image J software, and the degree of cuticular darkness was scored as luminance intensity on a grey scale between 0 and 255 (0 darkest, 255 brightest). The weighted average luminance was measured for each specimen on the beetle elytra in duplicate and expressed as the mean of two technical replicates.

### 2.6. Hemolymph Sampling, Total Hemocyte Counts, and Phenoloxidase Enzymatic Assay

To assess the effect of exposure on immunocompetence, the hemolymph was collected from the beetles 2 and 7 days after initial exposure to the commercial formulations of MTB and TG. The time points for performing the total hemocyte counts (THCs) and measuring the PO enzymatic activity were chosen according to the age of the beetles, i.e., young (2 days) and sexually mature (7 days), which also corresponded to the days of exposure to the treatments.

Males and females of the treated (MTB and TG) and control groups were punctured ventrally at the pro-mesothorax junction with a 29-gauge needle to collect hemolymph, which was used in the following protocols.

For THCs, 3 μL of hemolymph was collected and diluted 1:4 in cold sterile phosphate buffer (PBS, 10 mM; Sigma-Aldrich, Milan, Italy) at 4 °C, supplemented with 17 mM EDTA (Ethylenediaminetetraacetic acid disodium salt dihydrate, Sigma-Aldrich) to prevent clot formation. Briefly, 10 μL of the PBS–hemolymph solution was loaded into the Bürker chamber (Carlo Erba, Milan, Italy) and hemocytes were counted under a light microscope (LM) at 100× magnification (Zeiss Primo Star). THCs are expressed as the number of cells (mean ± SE (standard error)) per mL of hemolymph.

The enzymatic activity of plasmatic phenoloxidase (PO) was measured spectrophotometrically as dopachrome formation from 3,4-dihydroxy-L-phenylalanine (L-DOPA). In each plate, cell-free hemolymph from different experimental groups was analyzed in parallel to evaluate both activated basal and total (including activating pro-enzyme) PO. Hemolymph (4 μL, 15 individual replicates for each experimental condition) was collected as described above, immediately diluted to 1:15 with ice-cold sterile PBS at 4 °C, and centrifuged at 10^4^ rpm for 5 min at 4 °C. The supernatant (cell-free hemolymph–PBS mixture) was collected and stored at −20 °C. To evaluate total PO, the zymogen was activated by adding 10 μL of LPS (lipopolysaccharides from Escherichia coli O127:B8; Sigma-Aldrich, Milan, Italy) (1 mg mL^−1^ in cold sterile PBS) to 10 μL of supernatant in the well of a sterilized 96-well microtiter plate and incubated for 5 min at room temperature, as described in (Cavaliere et al., 2019). To measure basal PO, 10 μL of cell-free hemolymph was instead added to each well and mixed with 10 μL PBS (the vehicle used to dilute the LPS activator) 80 μL L-DOPA (3-(3,4-dihydroxyphenyl)-L-alanine; L-3-hydroxytyrosine, Sigma-Aldrich; 3 mg mL^−1^ in PBS) was added to each well. The blank was loaded with the same volume of PBS in place of the cell-free hemolymph. The change in absorbance was recorded at 492 nm and 25 °C for 60 min at 15 s intervals using a plate reader (Thermo Scientific Multiskan FC). All samples were analyzed in duplicate and expressed as the mean of two technical replicates. Enzyme activity was measured as the slope (absorbance vs. time) of the reaction curve during the linear phase of the reaction (Vmax value, considering 15 min after the start of the reaction). The slope of the reaction curve at the Vmax value was plotted as the absorbance change per minute (ΔA_492_ min^−1^) for the samples in each group.

### 2.7. Statistical Analyses

Statistical analysis of element concentrations in insects was performed according to the different types of exposure (chemicals, time) and/or sex of the specimens using Statistica 7.1 package (Milano, Statsoft Inc.) and IBM SPSS 25. The accumulation of elements from herbicide (MTB) and fertilizer (TG) treatments was statistically analyzed as two separate data sets (C vs. TG; C vs. MTB). Males and females were analyzed as control and treated samples, considering treatment exposure periods of 2 and 7 days, respectively. The normality of the data was examined using the Shapiro–Wilk test. Analysis of variance (ANOVA) with Tukey HSD adjustment for multiple comparisons was used to identify groups with significantly different elemental concentrations (*p*-value < 0.05). Principal component analysis (PCA) was used to examine the variability among the data and to identify the presence of patterns, while factorial analysis (FA) was used to better explore the presence of latent factors driving differences among the considered samples [99,100,101]. Factors were extracted by principal axis factoring with varimax rotation, and factors with eigenvalues greater than 1 were considered significant. Biomagnification factor (BMF) was estimated under laboratory conditions and calculated as the ratio between the metal concentration in the beetle bodies (μg g^−1^, dry weight) from the control group and the diet (μg g^−1^ dry weight of wheat bran) or from treated groups and administered MTB and TG treatments, respectively [57].

R version 3.0.1 software [102] was used to evaluate the statistical significance of immunocompetence markers (THCs, total and basal enzyme activities), body mass, and cuticular darkness. These data did not show a normal distribution and homogeneity of variance (*p*-value < 0.05) and were compared using a Kruskal–Wallis test followed by pairwise comparisons using the Wilcoxon rank-sum test with Bonferroni correction. On the other hand, because relative mass loss data showed a normal distribution and homogeneity of variance, parametric one-way ANOVA was performed followed by Tukey HSD adjustment for multiple comparisons. Results are presented as mean ± standard error.

## 3. Results

### 3.1. Element Concentration in NPK Fertilizer, Metribuzin-Based Herbicide, and Wheat Bran

Different trace elements occurred in the wheat bran, fed to the beetles during the exposure experiments, and in MTB and TG commercial formulations (Table S1). The order of accumulation for elements exceeding 1 µg/g was as follows:

(a) K > Ca > Mg > Na > Fe > Al > Cr > Mn > Sr > Rb > Zn > V > Y > U > La > Ni > Cu > Ba > Ce > Nd > Ga > Cd > Sc > As > Yb in fertilizer, (b) Al > Na > Mg > Fe > K > Ca > Be > Se > Bi > V > In > Ce > Ga > Ba > Zn > Mn > Ni = Cu > Sr > La > Nd > Sc in herbicide, and (c) K > Mg > Zn > Fe > Na > Mn > Ca > Cu > Rb > Al > Ba > Sr in wheat bran.

### 3.2. Element Accumulation in T. molitor

The concentrations of the elements in the beetles of the groups treated with MTB and TG and the controls are shown in Tables S2 and S3, respectively. Li, Sc, In, and Th were excluded because they were not detected in the tested samples.

**Figure 2:**
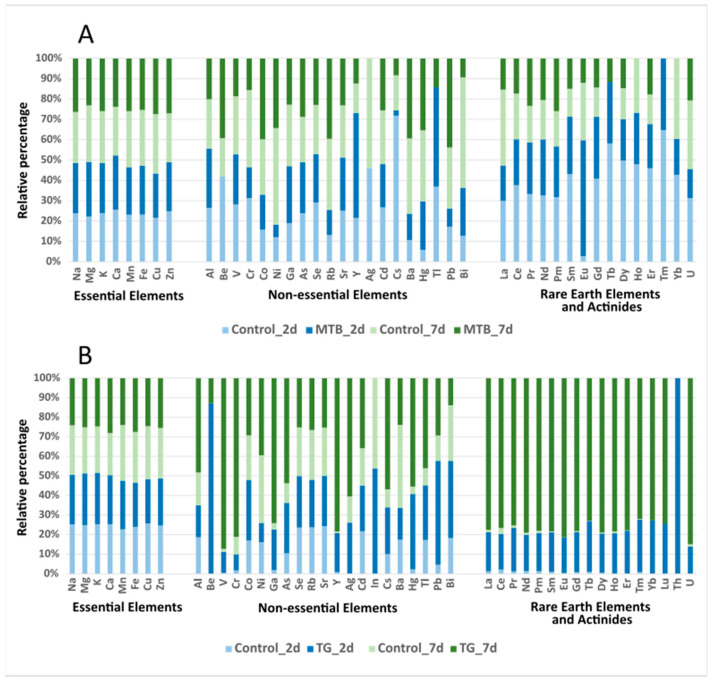
Relative abundance of elements in *T. molitor* adults. The relative percentages of each element refer to the respective experimental groups of beetles exposed to metribuzin-based herbicide (MTB, **A**) and NPK-based fertilizer (TG, **B**), and the control group, at 2 (2 d) and 7 (7 d) days of treatment.

The most abundant elements recorded in the beetles of both the control and treated groups were K > Na > Mg > Zn > Fe > Ca > Cu > Mn > Al, while the other identified elements accumulated in different orders that varied with treatment, time, and sex, as shown below (Tables S2 and S3; Figure 2).

In the group exposed to the MTB herbicide, the order of the element accumulation was Sr > Hg > Rb > Ba > Se > Ni > Cd > Cr > Co > V > Ga > Ce > Y > Nd > La > Pm for the females and Hg > Sr > Rb > Ba > Se > Cr > Ni > Cd > Pb > Co > Y > As > Ce > Bi > V in the males after 2 days of the initial exposure, whereas it was Hg > Sr > Rb > Ba > Ni > Se > Cr > Pb > Cd > Co > V > Ga > Ce > Y > La = Nd = Pm in females and Hg > Sr > Rb > Ba > Ni > Se > Co > Pb > As > Ce > La > Pm > = Bi in males at day 7 from the initial exposure (Table S2; Figure 2A).

Analysis of variance showed that Cr was the only element that did not show significant differences (*p*-value > 0.05) among the sampled groups. In females, significant increases in Na, Mg, K, Ca, and Ga concentrations were recorded in the MTB-treated samples compared to the controls after 2 days, while the concentrations of Mg, Ni, Ga, As and La were lower in the MTB-treated group, and Pb and Rb increased after 7 days of exposure. On the other hand, in males, a significant increase in Eu was recorded at 2 days, but a decrease in Na, K, V, Cd, La, Ce, Ho, Er, Yb, Hg, and U occurred in the MTB-treated group compared to the control one. After 7 days of exposure, the concentrations of V, Ga, As, Ho, Yb, Hg, Bi, and U were lower in the MTB-treated group than in the control one, while the concentrations of Co, Zn, Ba, and Pm were higher in the MTB-treated group than in the control ones. Exposure to the herbicide over time resulted in an increase in Ni, Rb, Ba, and Pb concentrations and a significant decrease in Mg, Ca, Sm, and Gd in MTB-treated females. The concentration of Na, K, Mn, Fe, Co, Ni, Cu, Zn, Rb, Cd, Ba, and Pb was higher in males treated for 7 days than in those treated for 2 days, while the accumulation of Al, Y, Eu, Ho, Tm, Yb, and Tl in the samples decreased with time. Gender-related differences in element accumulation were observed at both time points. In general, females accumulated higher levels of Mg, K, Sr, Se, Ga, Nd, and V than males, while Cu, Co, Zn, Cd, As, Sr, Hg, and U accumulated higher levels in males than females. Although the concentrations of Na, Ca, Mn, Fe, Ba, Pm, Sm, and Gd were higher in females than in males after 2 days of exposure, while Tm, Yb, Tl, and Bi were higher in males, no differences were found between males and females for these elements after 7 days of treatment (Table S2).

In beetles exposed to the TG fertilizer, 2 days after the initial exposure (Table S3; Figure 2B), the order of metal accumulation was as follows:

(a) Sr > Rb > Ba > Hg > Se > Ni > Y > Cd > U > V > Co > Ce > As > La > Pm > Cr > Nd > Ga > Gd > Yb > Sm > Er > Cs in females, and (b) Sr > Hg > Rb > Cr > Ba > Se > Cd > Ni > Co > Y > U > V > Pb > As > Ce > La > Pm > Nd > Be > Ag > Ga=Pr in males. Seven days after the initial exposure, the ranking of element accumulation was Sr > Hg > Cr > Rb > Ba > Ni > V > U > Y > Se > Cd > Ce > La > Pm > Nd > Co > Ga > Pb > Gd > Dy > Sm = Ho > Yb in females and Sr > Cr > Rb > Hg > Ba > Ni > V > U > Se > Y > Cd > Ce > La > Pm > As > Co > Nd > Ga > Pb > Ag > Dy > Gd > Yb > Sm > Er > Ce > Ho > Tb > Eu in males. Analysis of variance showed significant differences in the accumulation of elements among all the tested groups (*p* < 0.05). In females, a significant accumulation of some elements was observed in the TG-treated group compared to the control one for Na, Be, Mg, K, Mn, Co, Ga, As, Rb, Tb, Lu, and Tl after 2 days and for Al, Ca, V, Cr, Ga, As, Y, Cd, La, Ce, Pr, Nd, Pm, Sm, Gd, Dy, Ho, Er, Hg, and U 7 days after the initial exposure. In males, the accumulated elements were Be, Mg, Ca, Co, Cu, Se, Rb, Ag, In, Hg, Pb, Bi, and Th at 2d, while Al, Ca, V, Cr, Mn, Co, Ga, As, Y, Ag, Cd, In, Cs, Ba, La, Ce, Pr, Nd, Pm, Sm, Eu, Tb, Dy, Ho, Er, Tm, Yb, Lu, Hg, Tl, Bi, and U increased 7 days after the initial exposure compared to the control group.

The levels of Al, V, Cr, Fe, Zn, Ga, As, Y, Cd, La, Ce, Pr, Nd, Pm, Sm, Gd, Dy, Ho, Er, Hg, Pb, and U increased significantly over time in the treated females, while Na, Mg, and Be decreased. In males, TG fertilizer exposure caused a significant accumulation of Al, Be, V, Cr, Fe, Ga, As, Y, Ag, In, Cs, Ba, La, Ce, Pr, Nd, Pm, Sm, Eu, Gd, Tb, Dy, Ho, Er, Tm, Yb, Lu, Tl, Pb, Th, and U.

Differences in element accumulation were observed between the sexes. Two days after the initial exposure, the concentration of Mg, Mn, Rb, and Sr was higher in females than in males, while males accumulated Be, Co, Zn, In, and Pb. At 7 days after the first exposure, males accumulated more Na, K, Co, Ga, Cs, La, Eu, Tb, Tm, Yb, Lu, and Tl than females, while females accumulated more Al and Hg than males (Table S3).

**Figure 3:**
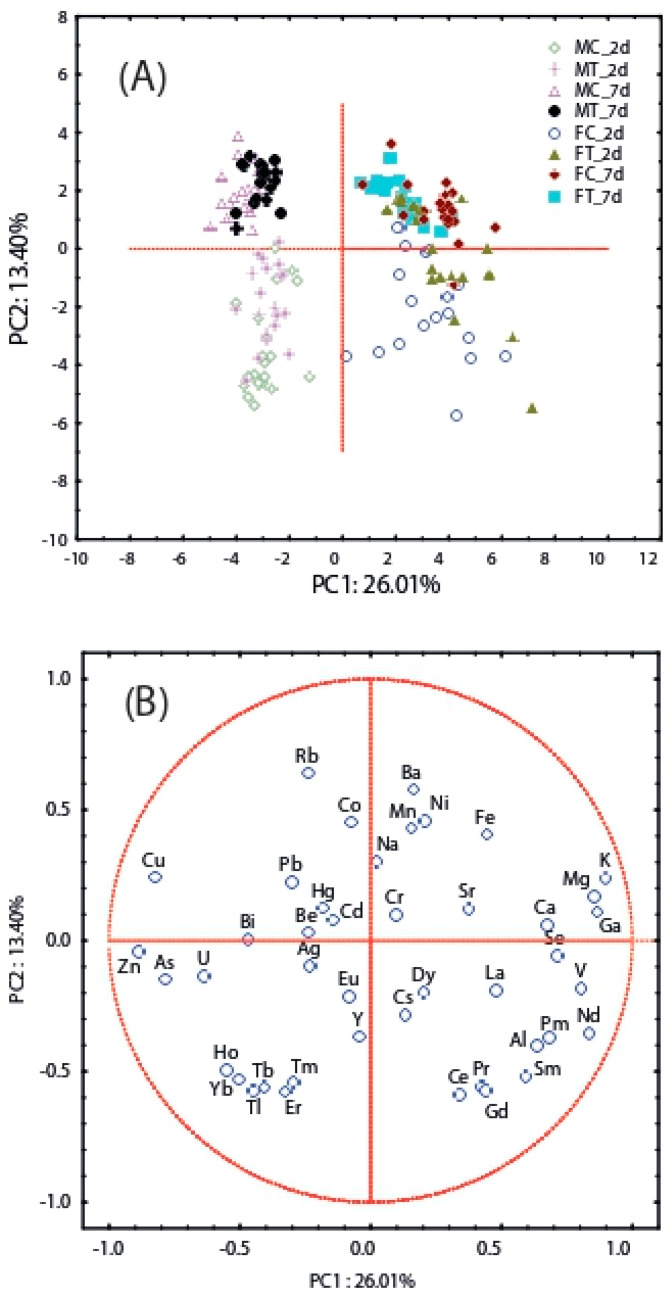
Principal components analysis: Scores and loadings plots on the first two PCs for the treated *T. molitor* adults with the metribuzin-based herbicide (**A**,**B**).

The datasets of element concentrations in beetles were explored by a principal component analysis (PCA) to detect the presence of patterns among samples from exposure tests in an unsupervised mode. The MTB-treated samples and corresponding control ones showed a clustering on PC1 according to their sex (Figure 3A,B) (i.e., males and females had negative and positive scores on the first principal component PC1, respectively). The elements with the most positive loadings (>0.7) on PC1 were, in decreasing order, K, Ga, Mg, Nd, V, and Se; they predominantly characterized the female specimens. On the other hand, As, Cu, and Zn were the elements with markedly negative loading values (<−0.7) on PC1 that characterized males (Figure 3A,B). The metals leading PC2 can be influenced by the exposure time. Indeed, no separate patterns over time were found in the treated and control groups, for both males and females. On the contrary, separation by age was observed in males, evident as a clustering of specimens between 2-day (young)- and 7-day (sexually mature)-exposed beetles (Figure 3), unrelated to treatment.

**Figure 4:**
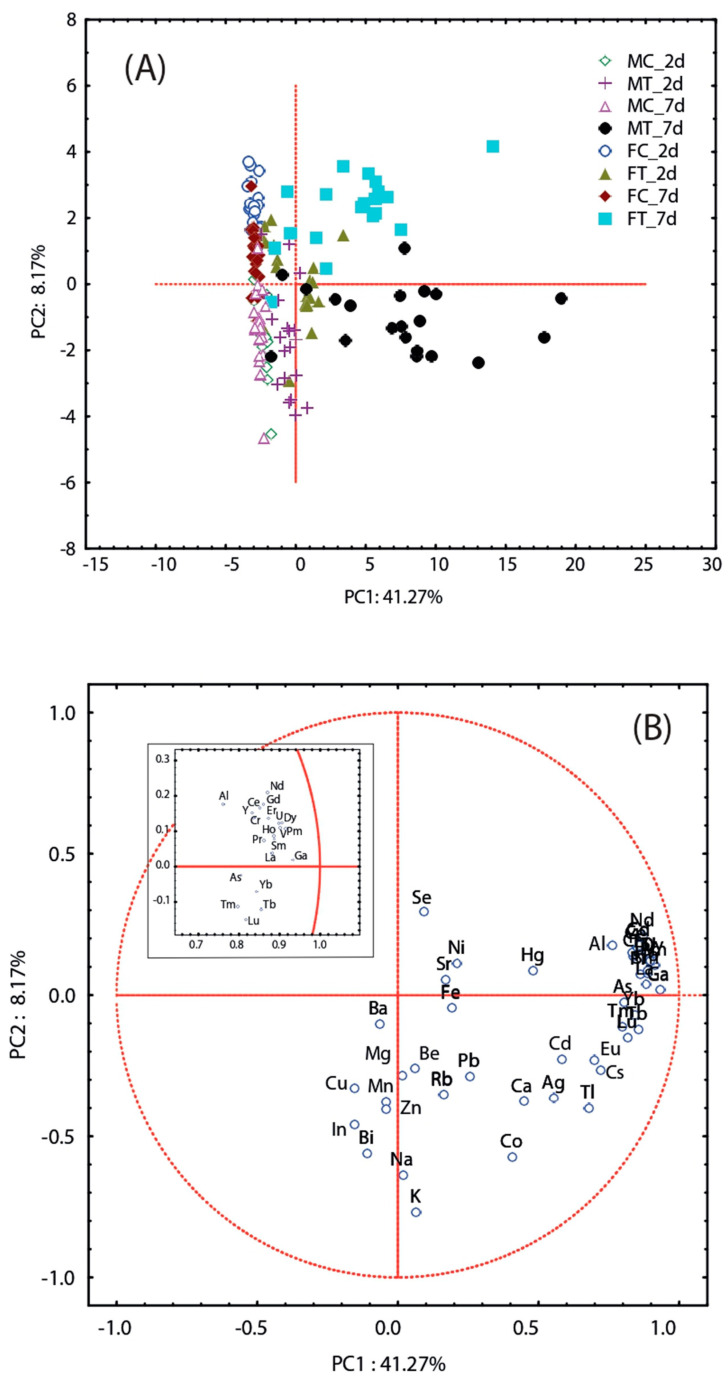
Principal components analysis: Scores and loading plots on the first two PCs for the T. molitor adults treated with NPK fertilizer (**A**,**B**).

PC1 explained 41.27% of the variation of samples exposed to NPK fertilizer as a function of treatment time and was mainly driven by Al, V, Cr, U, and REEs (Figure 4A,B). With increasing fertilizer exposure, the samples tended to shift to increasingly positive values for PC1, highlighting the difference between the treated samples. The metals driving PC2 may be influenced by sex. Indeed, the male samples had negative values, and the female samples had positive values.

A study of latent factors carried out by principal axis factoring with varimax rotation on the dataset of MTB-treated samples allowed the extraction of 10 factors explaining a cumulative variance of 72.9%. The elements with higher loadings (>0.5) on the first factor were Pm, Nd, Sm, Pr, Gd, V, Al, Ce, and La (Table S4). The same investigation performed for the dataset of TG-treated samples resulted in the extraction of eight factors with a cumulative variance of 72.2% (Table S5). In this case, the elements that loaded together were Nd, Al, Ce, Gd, Dy, Y, V, Ho, Sm, Cr, U, Pm, Er, As, La, Hg, Ga, and Pr. Again, the first factor is characterized by elements belonging to both light and heavy REEs. In addition, these are Al and V, as already observed in MTB herbicide treatment, and toxic elements such as Hg, U, As, and Cr.

### 3.3. Biomagnification Factor

Adults of *T. molitor* had significant concentrated amounts of Cu and Zn, regardless of the treatment (Table 1). The biomagnification of Na, Cu, Zn, Se, and Cd was observed over time in both sexes for the control group. Moreover, a dimorphism was observed in the BMF values of Cr (higher in females), and Bi and U (higher in males). The ingestion of TG induced the biomagnification of Cu and Zn, while exposure to MTB leads to the biomagnification of K, Mn, Cu, and Zn.

### 3.4. Body Conditions

**Figure 5:**
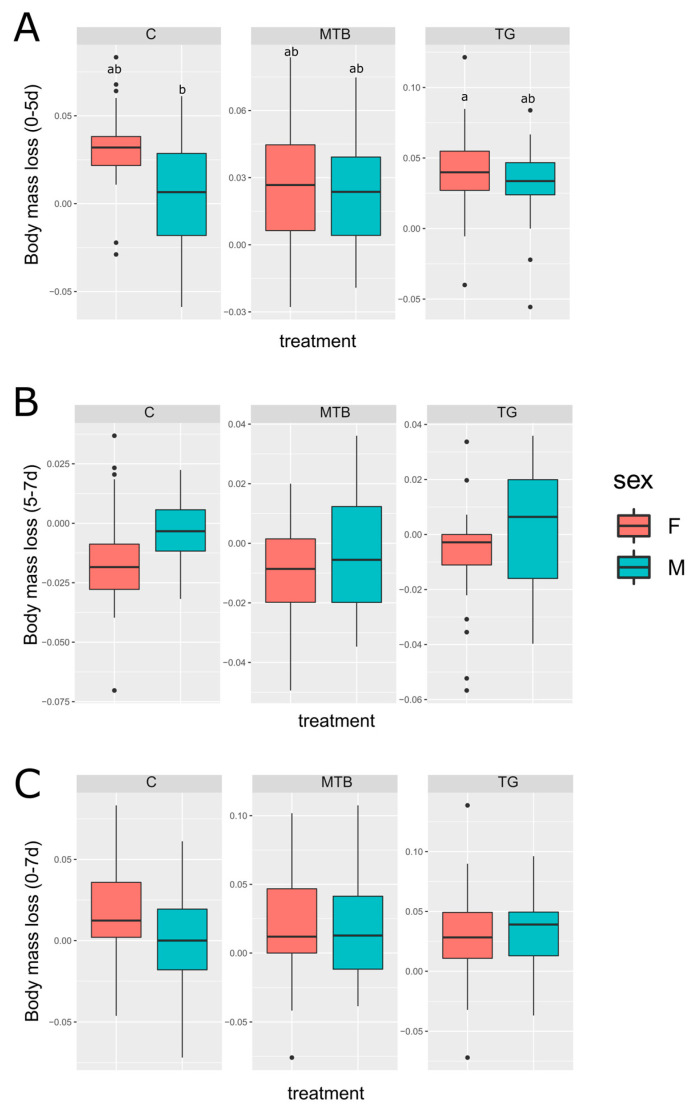
Relative loss of body mass in *T. molitor* exposed to metribuzin-based herbicide (**MTB**; N = 30) and NPK fertilizer (**TG**; N = 30) and in the control group (**C**; N = 30). Panels show relative body mass loss in males (M) and females (F) between 0 and 7 (**A**), 0 and 5 (**B**), and 5 and 7 (**C**) days after eclosion. Boxplots not sharing the same letter are significantly different at *p*-value < 0.05. Where letters are not shown, no significant differences were found.

In the first 5 days after eclosion, positive relative mass loss was recorded in all experimental groups, indicating a slight reduction in body mass of about 3%. However, a recovery occurred 7 days after the eclosion, with a negative relative mass loss, although the gain was less than 1% of body mass. When comparing values over the entire treatment period from 0 to 7 days, no significant differences in the relative mass loss were found between males and females (*p* > 0.05, Figure 5A). However, when considering the interval from 0 to 5 days post-eclosion (Figure 5B), a trend was observed between males and females in the control group (*p* = 0.06), suggesting a possible influence of sex on the loss of body mass during maturation, with the loss being greater in females (3.08 ± 0.60%) than in males (0.41 ± 0.72%). On the contrary, males (MTB: 2.19 ± 0.54%; TG: 3.15 ± 0.69%) showed a comparable mass loss to females (MTB: 2.79 ± 0.69%; TG: 3.95 ± 0.75%) within 5 days after the eclosion of treated beetles. A trend was also observed between control and TG-exposed males (*p* = 0.05), showing a greater loss in the TG group. There was also a significant difference between females in the TG group and control males (*p* < 0.05). No significant differences were found between the control, TG, and MTB-treated groups when assessing relative mass loss between 5 and 7 days after eclosion (*p* > 0.05, Figure 5C).

### 3.5. Total Hemocyte Counts

**Figure 6:**
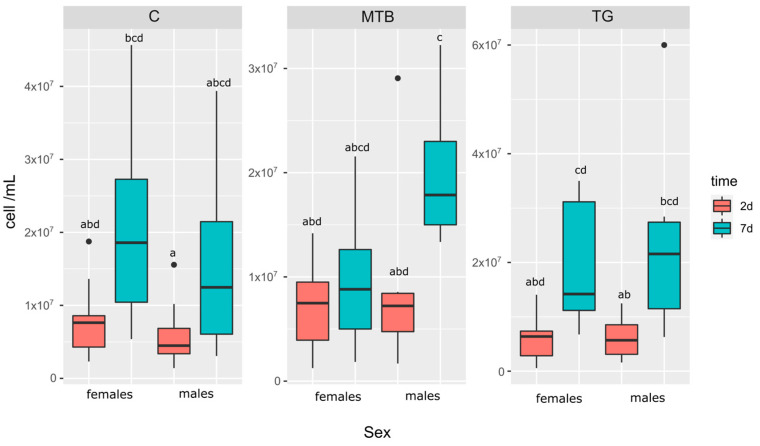
Total hemocyte counts (THC) expressed as cell mL^−1^, in *T. molitor* exposed to metribuzin-based herbicide (MTB; N = 11) and NPK fertilizer (TG; N = 11) and in the control group (C; N = 11). Panels show the density of circulating hemocytes in females and males at 2 and 7 days post-eclosion. Boxplots not sharing the same letter are significantly different at *p*-value < 0.05.

Hemocyte density was found to be dependent on the age of the beetle (Figure 6). Indeed, an increase in circulating hemocytes was recorded 7 days after eclosion (males: (15.14 ± 3.74) × 10^6^ cells mL^−1^; females: (20.63 ± 4.71) × 10^6^ cells mL^−1^) compared to THC in 2-day-old beetles (males: (5.71 ± 1.13) × 10^6^ cells mL^−1^; females: (7.77 ± 1.35) × 10^6^ cells mL^−1^) in the control group. Although 7-day-old females and males showed an approximately threefold increase in THC over time, the change was not statistically significant (*p* > 0.05), indicating a greater individual availability of circulating cells in the hemocoel in both males and females after 7 days. The same trend was observed in beetles from the TG-treated group. Although circulating hemocytes increased in 7-day-old specimens (males: (19.86 ± 3.52) × 10^6^ cells mL^−1^; females: (22.22 ± 4.56) × 10^6^ cells mL^−1^), there was no statistically significant difference with the corresponding younger groups (2-day-old *p* > 0. 05; males: (5.57 ± 1.10) × 10^6^ cells mL^−1^; females: (6.00 ± 1.15) × 10^6^ cells mL^−1^), although a trend (*p* = 0.06) was reported comparing TG-treated females at 2 days with those at 7 days. In addition, TG-treated males showed a significant change at 7 days after eclosion compared with young (2 days) males from the control group (*p* < 0.05), indicating a greater increase over time in males from the TG-treated group. In the male group exposed to MTB, the increase in THC was greater after 7 days ((20.10 ± 1.96) × 10^6^ cells mL^−1^), showing a statistically significant change (*p* < 0.05) compared with the 2-day-old beetles (Figure 6, MTB; (8.17 ± 2.21) × 10^6^ cells mL^−1^). The cell density of MTB-treated males at 7 days was also significantly higher (*p* < 0.05) than the values found in all groups of young males and females of the control and the TG- and MTB-treated groups. The MTB-treated females did not follow the same behavior as the other groups; rather, the circulating cells remained stable over time (*p* = 1; 2d: (7.29 ± 1.29) × 10^6^ cells mL^−1^; 7d: (9.76 ± 2.35) × 10^6^ cells mL^−1^), showing the lowest density of circulating hemocytes after 7 days. No significant gender differences in THC were found in any of the groups tested (*p* > 0.05).

### 3.6. Plasmatic Phenoloxidase Enzyme Activity

**Figure 7:**
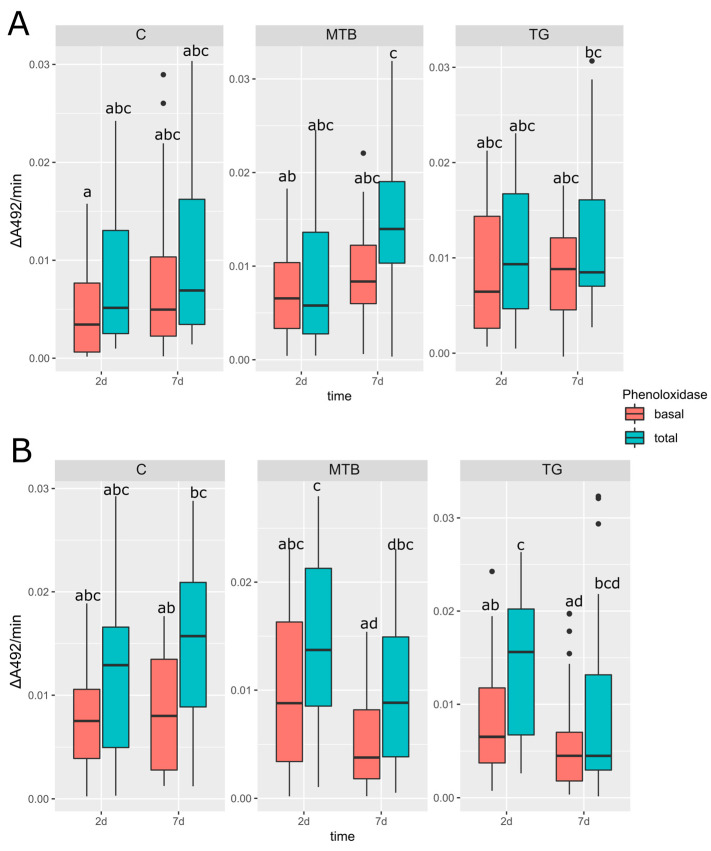
The plasmatic basal and total phenoloxidase enzyme activities (PO) in untreated (C; N = 15) *T. molitor* adults and treated ones with the metribuzin-based herbicide (MTB; N = 15) and NPK fertilizer (TG; N = 15). The enzyme activity was recorded as absorbance units per min (ΔA_492_ min^−1^) in females (**A**) and males (**B**) at 2 and 7 days after eclosion. Boxplots not sharing the same letter are significantly different at *p*-value < 0.05.

Analysis of plasmatic PO enzyme activity in the control group showed no statistically significant difference in relation to sex and age for both basal and total enzyme activity (*p* > 0.05) (Figure 7A,B). However, a slight increase in total PO activity was observed in 7-day-old males (Figure 7B), while total plasma PO enzyme levels were lower and more stable in females than in males at both time points (Figure 7A). This slight variation was evident only in activated zymogen activity and not in basal plasma phenoloxidase activity. No differences were found between basal and total phenoloxidase in either sex (*p* > 0.05), although total PO enzyme activity was higher than basal in males.

In the MTB- and TG-treated groups, no significant differences (*p* > 0.05) were found when comparing basal and total PO enzyme activity with control group values in both males and females. However, although not statistically significant (*p* > 0.05), the treatments reduced the plasmatic total PO enzymatic activity levels in both MTB- and TG-treated males compared to the young beetles in each group after 2 days. Furthermore, MTB- and TG-treated groups showed an inverse response to the 7-day control group, where the stability of total PO enzymatic activity was observed over time. A significant difference (*p* < 0.05) was observed between total and basal PO enzymatic activity in TG-treated males 2 days after eclosion, but not in MTB-treated males, although basal PO enzymatic activity was lower than total PO enzymatic activity. The same trend is not evident in females, as they showed lower levels of total PO enzyme activity comparable to basal PO activity.

### 3.7. Cuticular Darkness

**Figure 8:**
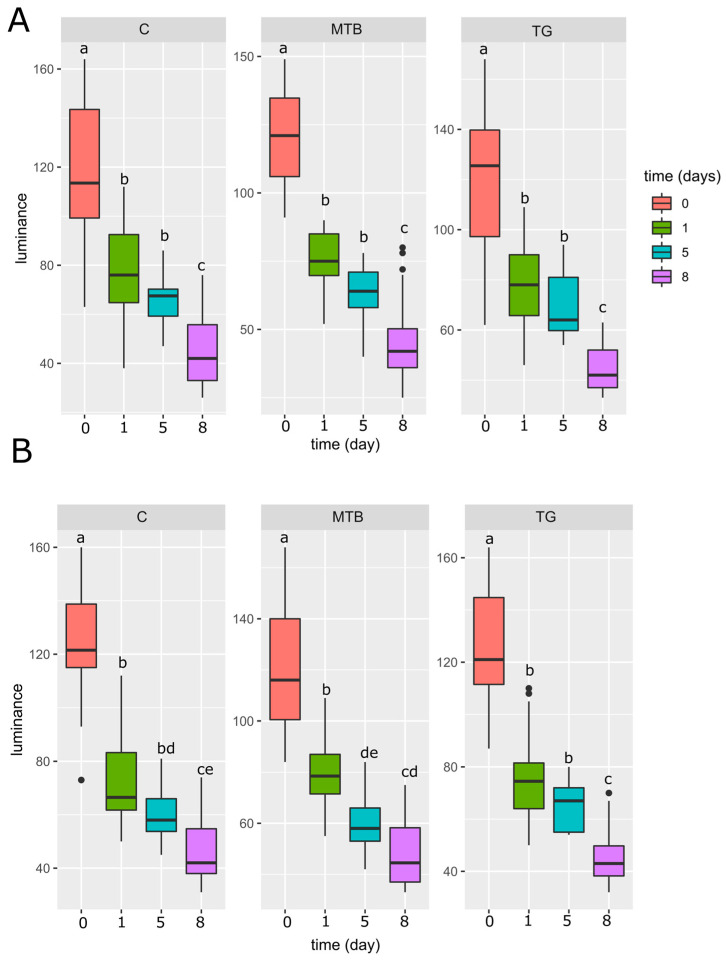
Cuticular darkness of elytra, expressed as luminance degree, in adult *T. molitor* exposed to the metribuzin-based herbicide (MTB; N = 30) and NPK fertilizer (TG; N = 30) and in the control group (C; N = 30). Cuticular darkness is shown in females (**A**) and males (**B**) at 0, 1, 5, and 8 days after eclosion. Boxplots not sharing the same letter are significantly different at *p*-value < 0.05.

A clear time-dependent relationship was found for cuticular darkness, resulting in a decrease in luminance intensity over time proportional to cuticular darkening. In all experimental groups, regardless of sex (Figure 8A,B), there was a significant change (*p* < 0.001) at the transition from day 0 to day 1, showing a rapid change in elytra. The decrease 5 days after eclosion was not statistically significant in the control group and TG-treated beetles, in both females (Figure 8A) and males (Figure 8B), and in MTB-treated females (*p* > 0.05), whereas a statistically significant reduction in luminance was recorded in MTB-treated males 5 days after eclosion (*p* < 0.01; Figure 8B) compared with the value recorded on day 1. The variation between the luminance recorded on day 5 and day 8 was statistically significant in the control and TG-treated beetles, both males (*p* < 0.001) and females (*p* < 0.001), and in MTB-treated females (*p* < 0.05), while no statistically significant differences were found in MTB-treated males (*p* > 0.05). This suggests that darkening reached a plateau earlier than in the other groups, as confirmed by the absence of significant differences (*p* > 0.05) between the values recorded at 5 days in the control males and those of MTB at 8 days after eclosion (Figure 8B).

## 4. Discussion

### 4.1. Trace Elements in Metribuzin-Based Herbicide and NPK Fertilizer

Our results show that trace elements are present in a tested metribuzin-based herbicide and NPK fertilizer, as reported for other commercial formulations in previous studies [30,31,45]. Non-essential heavy metals, such as Cd, Pb, Cr, and Al, and essential Cu, Fe, Mn, Co, Zn, and Ni were found in both the herbicide and NPK blends. The concentrations of Ni, Pb, As, and Cd and the relative percentages of Mn (0.1–0.5%), Fe (0.5–2%), Co (0.1–0.02%), Cu, and Zn (0.1–0.5%) were within the values allowed by the EU regulation for fertilizers, while Cr was the only element to exceed the limit of the tolerated concentrations (2 µg g^−1^) [10]. However, the concentrations of Al, Ba, Be, Cr, Co, In, Fe, Li, Ni, Se, Ag, Sr, Tl, V, and Zn were above the limits based on human toxicity data provided by national and international agencies [31]. For the metribuzin-based herbicide, the high Al content may have been included in the formulation by the manufacturers as an excipient to allow the penetration of the active ingredient into the target plant tissue and to increase its toxicity, because it acts as a root growth inhibitor [103]. Although the environmental risk may be negligible due to the concentration of most elements per kg, the repeated application of herbicides and fertilizers over an extended period of time per hectare in conventionally or integrated cropped areas results in a pervasive, emerging problem of trace element accumulation in agricultural soils [45,104,105]. As a result, when concentrations exceed physiologically tolerable limits, adverse effects on the functional diversity of the soil ecosystem occur. For example, heavy metal residues cause side effects on microbial communities involved in biogeochemical cycling [106], interfere with plant metabolic processes, reducing their growth and germination [107], and accumulate in vegetables [108], posing a potential risk to human health [55,109]. Furthermore, 15 REEs were present in the tested commercial formulations, with Y, La, and Ce being the most abundant in the NPK blends, suggesting that not only fertilizers [67] but also herbicides contribute to the accumulation of REEs in soils of agricultural areas worldwide.

### 4.2. Accumulation and Biomagnification of Trace Elements in Males and Females of T. molitor

The field concentrations tested in our study result in the accumulation of essential, nonessential, and REEs in *T. molitor*, confirming the hypothesis of potential exposure risk from ingestion by species inhabiting the soil of croplands and consequent accumulation and transfer of elements in the trophic web, as noted in our previous study [58]. Unsupervised chemometric analysis revealed that the data were structured into patterns that corresponded to different groups of insects based on their gender and the duration of treatment exposure. FA also demonstrated how the variability in most of the trace elements could be attributed to the first latent variable, which can be associated with the treatment given to the exposed insects (Tables S4 and S5). The accumulation pattern of trace elements in insects depends on age, sex, physiological status, life stage, and trophic level, as well as the concentration of an element in the diet or environment [110,111,112,113]. Although we used beetles of the same age to show variation in the accumulation of elements over time in both females and males according to treatment, high standard deviation values were observed for several elements, and the differences in accumulation patterns in both treated groups indicated a large variability in individual intake by ingestion during the exposure period. In general, our results showed that Na, Mg, K, Ca, Cu, Mn, Fe, and Zn were the essential elements that accumulated most in *T. molitor* females and males during the exposure period. The homeostatic regulation of trace element concentrations in insects depends on the ability to balance absorption and excretion rates and detoxification mechanisms. Although there is wide variability among insect species, excess elements are mainly sequestered by metal-binding proteins (e.g., metallothioneins) [114] or by incorporation into inorganic crystalline concretions [92,115] in the epithelium of the midgut and Malpighian tubules [116,117,118,119] or excreted [120,121] with a reduction in their toxic effects. Metals acquired through the oral ingestion of contaminated food accumulate with increasing concentrations from the intestine to the Malpighian tubules, as has been demonstrated in the soil-dwelling beetle *Pterostichus oblongopunctatus* [122]. The metabolic patterns and metallothioneins involved in the regulation of the homeostasis of essential (Fe, Zn, and Cu) and nonessential metals (Ni, Hg, Pb, and Cd) have been studied in *Drosophila melanogaster* [123]. Metals such as Cd, Pb, and Zn are sequestered in various tissues such as the gut including Malpighian tubules, fat body, hemolymph, muscle, and exoskeleton, as described in larvae of *T. molitor* [124,125] and the gypsy moth *Limandria dispar* [126]. The elements accumulated in the cuticle are released during molting and metamorphosis, while the residues remain trapped in the muscles and fat bodies after eclosion, as found in mealworm beetles [127] and lepidopterans [128]. Therefore, we assume that the concentrations of some elements found in the 2-day-old control samples are residues resulting from accumulation in the larval stage. From an ecological perspective, this suggests that both laboratory and field studies of metal bioaccumulation in holometabolous insects from agricultural soils require analyses that include the larval and pupal stages to evaluate element transfer during developmental stages, as observed in the red mason bee [129].

Gender-related differences occurred in the uptake and accumulation of nonessential elements and REEs in *T. molitor*, and the accumulation pattern depended on their concentration in the herbicide and fertilizer administered. In the group exposed to NPK fertilizer, concentrations of elements such as Ag, Cs, Eu, Yb, Lu, Tl, and Pb accumulated mainly in males, while Al, V, Cr, As, Se, Ni, Hg, U, and REEs (Ga, Y, La, Ce, Pr, Nd, Pm, Sm, Gd, Dy, Ho, Er) increased with the exposure time from immature to mature adults but did not depend on sex. Exposure to the metribuzin-based herbicide resulted in a different accumulation pattern from the group exposed to the NPK fertilizer. We found that females treated with MTB-based herbicide accumulated higher concentrations of essential elements (Na, Mg, K, Ca) than males, while the nonessential elements and REEs showed higher accumulation in 7-day-old mature males (i.e., Cu, Pb, Hg, Cd) and females (i.e., Cr, Si, Ga, Ni) than in 2-day-old immature subjects. Moreover, differences between sexes have been described in grasshoppers [130], *Drosophila melanogaster* [131], and lepidopteran *Spodoptera litura* [128] exposed to heavy metals, mainly affecting the activity of detoxifying metabolic enzymes. Differences in metal concentration patterns may reflect a sexual dimorphism in dietary requirements, which can result in sex-specific nutritional limitations and have ecological consequences in wild species exposed in the field. Therefore, further studies are required to examine toxicokinetic effects and physiological changes in females and males as a result of exposure to contaminants. In addition, given the toxic effects of metribuzin on non-target animals [35], it remains an open question whether the metribuzin-based commercial formulation impairs the ability of the biological compartment to sequester or release trace elements.

In the ecological risk assessment of exposure to trace elements, biomagnification shows that a specific element is accumulated above a certain threshold, resulting in increased bioavailability at a higher level of the food web. Exposure to the metribuzin-based herbicide increased the concentration and bioavailability of Cu (by 7- to 16-fold), Se (by 2- to 3-fold), Zn (about 30- to 60-fold), and Cd (about 3.5- to 10-fold) compared to the control group, indicating a higher potential for food chain transfer. This could be the result of a combination of conditions, such as the interaction between elements, the loss of the ability to remove and store elements, and/or the interference of the herbicide’s active ingredient with cell membrane transport systems. The biomagnification of these elements has also been noted in other invertebrates inhabiting soil in agricultural areas and feeding on organic matter, such as Diplopoda, Isopoda, and Collembola [59], or in secondary consumers such as carabid beetles [132]. This suggests that herbicides may act at the level of decomposers and predators by causing increased bioaccumulation of metal, which affects insect detoxification systems and has various sublethal effects on development, reproduction, pathogen response, and survival [61,92,133,134].

Although the mealworm beetle is known as a pest of stored food, it is also one of the most popular edible insects used as an alternative food and feed source [135,136,137] and has recently been approved for human consumption as a potentially suitable commission implement protein source [138,139]. The level of elements in the bran used to feed beetles in our study was extremely low and heavy metals were within the admitted limits (WHO/FAO, 2019). However, adults of *T. molitor* can concentrate nonessential elements such as Cr, Cd, Bi, and U over time, in addition to essential elements (Cu, Zn, Se). Thus, our results have highlighted a new concern related to the ability to accumulate and biomagnificate contaminants in this species derived from food sources such as wheat flour or vegetables used in livestock farming [140,141,142] and produced in conventionally managed crops with pesticides and fertilizers.

The exposure of males and females to the NPK fertilizer resulted in a significant accumulation of REEs, but there is little information on the biological role of these elements in animal systems [143]. Lanthanum replaces calcium in cellular transport channels by interfering with metabolism due to its chemical and physical properties [144]. In vertebrates, La, Ce, and Nd induced oxidative damage that accumulates in hepatocyte nuclei and mitochondria [145] as well as histopathological changes in the liver, lungs, and blood cells [146]. La has dose-related toxic effects on the survival and reproduction of soil invertebrates such as isopods, oribatid mites, and earthworms exposed to contaminated soils [71]. Moreover, the low biomagnification levels indicate a low potential for La to be transferred to the food web, as indicated by our laboratory results. However, monitoring studies have shown that the availability of REEs under field conditions has dose-dependent effects on soil macrofauna that promote or reduce community diversity [72] and pose risks to human health [147].

### 4.3. Exposure Effects on Physiological Parameters

Phenotypic traits such as cuticle melanization, immunological parameters, and mass loss are closely related to sex, nutritional levels, and aging in insects and are very plastic in overcoming environmental variability due to multiple biotic and abiotic factors [148,149], as have been studied in *T. molitor* [78] and other insect species [150].

Cuticle melanization is correlated with the levels of the amino acid tyrosine [149], and the same is true for immune factors such as phenoloxidase [148]. Thus, resource limitations and the resulting competition for use between different physiological processes can lead to trade-offs that cause phenotypic variation in cuticular color [151]. In *T. molitor*, cuticular melanization ceases to change after imaginal eclosion in the first week, independent of the final level of cuticular color, and the end of this period is coupled with sexual maturity [97]. Moreover, cuticle melanization is an indicator of investment in immunocompetence, affecting hemocyte density and phenoloxidase activity, both of which are higher in dark than in tan beetles [89,90]. In our study, the newly emerged beetles were fed ad libitum to ensure the constant availability of food, and we observed a rapid variation of the cuticle color over time in both males and females of the control group, in accordance with the literature [97]. Nevertheless, when monitoring the 5-day interval after eclosion, a greater mass loss was observed in the females. This could suggest a sex-dependent difference in resource allocation and metabolism, which undergoes profound changes during the transition from the immature to the mature stage. However, the sexual dimorphism observed in the control group in terms of body mass loss disappears in the specimens from the MTB- and TG-treated groups, suggesting that exposure interferes with metabolic processes and nutritional requirements affecting parameters such as cuticular darkness, phenoloxidase activity, and hemocyte density. Indeed, males exposed to the metribuzin-based herbicide were observed to darken more rapidly in the first 5 days after eclosion, suggesting premature browning and thus an early arrest of the process. However, this had no influence on the degree of melanization, which was reached 8 days after eclosion and for which no differences to the control group were found.

Sex differences in insect immunocompetence depend upon the trade-off resource allocation between reproduction and immune response [152]. We assume that the increase in circulating hemocytes observed at the transition to the sexually mature stage in both females and males of the control group protects against pathogens that might attack the organism at a stage when the cuticle is not yet fully melanized. However, exposure to the MTB-based herbicide resulted in a different response between the males, which increased circulating hemocytes, and the females, which kept them constant over time, suggesting a possible phlogistic effect. Although total PO enzyme activity was higher in young males than in females of the mealworm beetle, sexual dimorphism seemed to disappear in the MTB- and TG-treated groups as enzyme activity decreased over time. The effects of herbicides on insect immunity are an emerging concern for species involved in ecosystem services [23,153] causing side effects including the activation of pro-inflammatory and anti-inflammatory responses [154]. As metribuzin has been shown to affect immunological parameters in vertebrates, we assume that it caused an inflammatory state of the internal organs in *T. molitor* and reduced the density of hemocytes in favor of the recruitment of circulating cells, as observed in *Drosophila* [81,155]. In general, the onset of an inflammatory status is associated with increased oxidative stress [156], which impairs fertility and fitness. In the flour beetle, females can assess the immunocompetence of males based on pheromone signals and rely on this information for mate choice [157]. Similarly, females can discriminate males suffering from oxidative stress, as demonstrated in *T. molitor* males exposed to the herbicide paraquat [158]. The disruption of sexual selection was also observed in beetles exposed to atrazine, a herbicide of the triazine class [159]. Further studies should investigate which of the previously described cell populations [160] show an increase/decrease and whether a change in oxidative stress levels occurs over time in insects exposed to the field rate of herbicides and fertilizers to support our hypothesis.

Exposure to high concentrations of elements and the active ingredient of the herbicide not only impairs metabolic pathways for the removal and storage of contaminants but can also have effects on essential insect life traits, such as immunity, reproduction, and life span, by causing a physiological shift in energy allocation to maintain homeostasis [60]. The levels of circulating hemocytes and PO enzyme activity are generally very sensitive biological parameters for metal concentrations [161]. Essential and nonessential elements play dual roles in reproduction and immunity in insects [162,163]. For example, the exposure of grasshoppers to a high concentration of Zn (100–1000 µg g^−1^) during diapause causes a high frequency of apoptosis and necrosis in adult tissues and the aging of females evident in eggs laid, and also affects the duration of embryogenesis [164]. On the other hand, Zn increases phagocytosis and affects the activation and death of circulating hemocytes in *Musca domestica* and *D. melanogaster* [165]. Our results on Cu bioaccumulation in males upon exposure to the metribuzin-based herbicide could explain the large number of circulating cells at 7 days after eclosion. The response could implicate immunostimulation by Cu, as previously observed in insects [166] mollusks, and crustaceans [167,168]. Moreover, Cu is involved in PO activation through Cu-dependent tyrosinase activity [169]. However, a high concentration of this essential element resulted, upon the herbicide glyphosate exposure, in reduced PO activity and an altered melanization in *Galleria mellonella* and *Anopheles gambiae* [170].

## 5. Conclusions

This study highlights that metribuzin-based herbicide and a commercial NPK fertilizer formulation at concentrations commonly admitted in the field contain trace elements, including those nonessential to biological systems and REEs. Exposure experiments with *T. molitor,* simulating direct intake by ingestion, showed the resultant bioaccumulation of these elements with patterns varying by treatments. The application of an NPK blend fertilizer is the most consistent cause of REE accumulation over time, in addition to toxic elements (e.g., Sr, Hg, Cr, Rb, Ba, Ni, Al, V, U) that are also present in herbicide-treated beetles. Therefore, oral exposure can be expected to be the first route of metal uptake in insects inhabiting soil in agricultural areas where commercial formulations of fertilizers and herbicides are continuously applied, introducing trace elements into the soil. Moreover, the biomagnification factors for Cu, Zn, K, and Mn, as well as accumulation levels for other identified elements in *T. molitor*, suggest a high potential for transfer through the food web from species that feed on organic matter to predators in the agroecosystems. However, the accumulation of REEs is an emerging concern, and further studies are required to define their effect on animal biological systems, helping to define permissible regulatory limits in commercial formulations that do not harm organisms.

Sex-specific differences in element concentrations suggest that males and females are characterized by different uptakes, allocations, and excretions of elements, which ultimately indicates that each sex has specific demands for nutrients even in the period leading up to the reproductive phase. Although species-specific differences can occur, such information is crucial for predicting specific responses of wild insect species to realistic metal concentrations in the soil of croplands treated with agrochemicals such as herbicides and fertilizers. Therefore, further studies may clarify the differences between sexes in allocating their energetic resources to detoxification pathways in the polluted environment and how this may affect other energetic costly life traits such as reproduction and immunity.

The novelty in our study lies in measuring the physiological effects of exposure to the tested agrochemicals during the highly sensitive transition phase from newly emerged to sexually mature adults. Exposure to trace elements interferes with parameters such as melanization, cellular and humoral immune responses, and mass loss. This may indicate a disturbance of the metabolic pathways involving sequestration and detoxification in the maturation stages triggering a resource reallocation between sexual maturation and immune responses. Some elements of those identified are known to interfere with biological functions such as immune response. Higher Cu levels in males exposed to the metribuzin-based herbicide displayed an increase in circulating hemocytes. As a result, altered immune functions may have further implications on fitness.

The results obtained in this research lead us to consider that when assessing the risks of exposure to agrochemicals on wild species in the field, it must be considered that herbicides have trace elements in commercial formulations that, coupled with the active ingredient action, can interfere with non-target biological systems. Fertilizers also have harmful effects on soil organisms due to the accumulation of elements they bring to the soil, including essential elements if they exceed threshold values. Furthermore, physiological responses to agrochemicals and trace elements are conditioned by the age of organisms because of the allocation of time-varying resources to meet the challenge of survival while preserving reproductive fitness. All in all, soil testing and applying controlled-release fertilizers and mechanical weed control are two better practices suggested to preserve the biodiversity of wild insect species in the agroecosystem.

## Figures and Tables

**Table 1:** Biomagnification factors (BMFs) of metals in *Tenebrio molitor* females and males from control (CTRL), metribuzin-based herbicide (MTB), and NPK fertilizer (TG) groups.

Treatments						
	CTRL ^a^		MTB ^b^		TG ^b^	
	Females	Males	Females	Males	Females	Males
**Na**	**16.25–17.13**	**20.48–19.85**	0.09–0.09	0.07–0.09	0.68–0.59	0.62–0.65
**K**	0.57–0.61	0.81–0.68	**28.89–28.36**	**12.90–15.67**	0.02–0.02	0.02–0.02
**Mn**	0.08–0.10	0.10–0.13	**4.04–3.97**	**3.15–4.01**	0.07–0.06	0.05–0.06
**Cu**	**1.43–1.52**	**1.63–1.68**	**7.49–8.14**	**12.18–16.61**	**5.05–5.63**	**5.58–5.91**
**Zn**	**1.49–1.59**	**1.64–1.68**	**31.74–32.86**	**57.58–67.53**	**2.73–3.10**	**3.17–3.15**
**Se**	**3.05–2.53**	**1.70–2.47**	-	-	0.68–0.59	0.62–0.65
**Cr**	0.97–**5.28**	0.20–0.84	-	-	-	-
**Cd**	**1.15–1.53**	**2.49–1.67**	-	-	-	-
**Bi**	0–0.45	**1.41–3.95**	-	-	-	-
**U**	0–0.08	0.04–**2.44**	-	-	-	-

^a^ BMFs were calculated as the ratio between the metal concentration in beetle and food; ^b^ BMFs were calculated as the ratio between the metal concentration in beetle and administered treatment. Metal concentrations of food and MTB and TG commercial formulations are reported in Table S1 and for beetles in Tables S2 and S3. Each number pair indicates the value recorded for beetles 2 and 7 days old, respectively.

## Data Availability

The data presented in this study are available on request from the corresponding author.

## Supplementary Materials

The following supporting information can be downloaded at: https://www.mdpi.com/article/10.3390/toxics11060499/s1, Table S1: Mean concentrations and standard deviations of the investigated elements in wheat bran used to feed the beetles, metribuzin-based herbicide (Feinzin), and NPK fertilizer (Timagreen). Except where otherwise indicated (*), concentrations are expressed in µg/g. n.d.: not detected; Table S2: Elements accumulated in adults of *Tenebrio molitor* treated with the metribuzin-based commercial formulation. Mean concentrations and standard deviation of the analyzed elements for each group of beetles (20 samples for each group, results on a dry-weight basis). N.d.: not detected. (FC, female control; FT, female treated; MC, male control; MT, male treated; 2d, two days; 7d, seven days). n.d.: not detected; Table S3: Elements accumulated in adults of *Tenebrio molitor* treated with NPK fertilizer. Mean concentrations and standard deviation of the analyzed elements for each group of beetles (20 samples for each group, results on a dry-weight basis). N.d.: not detected. (FC: female control; FT: female treated; MC: male control; MT: male treated; 2d: two days; 7d: seven days). n.d.: not detected; Table S4: Principal axis factoring for metribuzin-based-herbicide-treated adults of *Tenebrio molitor*, matrix with factor loadings. Loadings below 0.5 were not considered to improve the understanding of the data structure; Table S5: Principal axis factoring for NPK-fertilizer-treated adults of *Tenebrio molitor*, matrix with factor loadings. Loadings below 0.5 were not considered to improve the understanding of the data structure.
