# Supplementary material for: Triazine Herbicide and NPK Fertilizer Exposure: Accumulation of Heavy Metals and Rare Earth Elements, Effects on Cuticle Melanization, and Immunocompetence in the Model Species Tenebrio molitor
Source: Toxics. 2023 Jun 1;11(6):499. doi: 10.3390/toxics11060499 (PMC10300948; doi:10.3390/toxics11060499)
Supplement: Supplementary file 1 [file toxics-11-00499-s001.zip › toxics-2390100-supplementary.pdf]

toxics

Article

Article

Triazine Herbicide and NPK Fertilizer Exposure: Accumulation of Heavy Metals and Rare Earth Elements, Effects on Cuticle Melanization, and Immunocompetence in the Model Species *Tenebrio molitor*

Attilio Naccarato [1], Maria Luigia Vommaro [2,*], Domenico Amico [3], Francesca Sprovieri [3], Nicola Pirrone [3], Antonio Tagarelli [1] and Anita Giglio [2,*]

[1] Department of Chemistry and Chemical Technologies, University of Calabria,87036 Rende, Italy; attilio.naccarato@unical.it (A.N.); antonio.tagarelli@unical.it (A.T.)
[2] Department of Biology, Ecology and Earth Science, University of Calabria, 87036 Rende, Italy
[3] CNR-Institute of Atmospheric Pollution Research, 87036 Rende, Italy; domenico.amico@iia.cnr.it (D.A.); f.sprovieri@iia.cnr.it (F.S.); pirrone@iia.cnr.it (N.P.)
* Correspondence: marialuigia.vommaro@unical.it (M.L.V.); anita.giglio@unical.it (A.G.); Tel.: +39-0984492982 (A.G.)

Citation: Naccarato, A.; Vommaro, M.L.; Amico, D.; Sprovieri, F.; Pirrone, N.; Tagarelli, A.; Giglio, A. Triazine Herbicide and NPK Fertilizer Exposure: Accumulation of Heavy Metals and Rare Earth Elements, Effects on Cuticle Melanization, and Immunocompetence in the Model Species *Tenebrio molitor*. *Toxics* **2023**, *11*, x. https://doi.org/10.3390/xxxxx

Academic Editor(s): Xiaoming Xia, Youhui Gong and Ting Li

Received: 24 April 2023
Revised: 27 May 2023
Accepted: 29 May 2023
Published: date

Copyright: © 2023 by the authors. Submitted for possible open access publication under the terms and conditions of the Creative Commons Attribution (CC BY) license (https://creativecommons.org/licenses/by/4.0/).

Supplementary material

Table S1. Mean concentrations and standard deviations of the investigated elements in wheat bran used to feed the beetles, metribuzin-based herbicide (Feinzin), and NPK fertilizer (Timagreen). Except where otherwise indicated (*), concentrations are expressed in μg/g. n.d.: not detected

Elements	Bran	Feinzin	Timagreen
Li	16.1 ± 2.5*	4.72 ± 0.40	0.5745± 0.0704
Be	1.1 ± 0.21*	86.7 ± 7.6*	0.5151 ± 0.0918
Na	108.1 ± 4.5	21667 ± 1832	5587 ± 853
Mg	2349 ± 49	5510 ± 470	6638 ± 1010
Al	6.84 ± 0.31	29169 ± 4877	2937 ± 706
K	7714 ± 281	397 ± 49	234348 ± 36448
Ca	94.7 ± 4.4	73.5 ± 4.9	19789 ± 1656
Sc	n.d.	1.141 ± 0.079	1.81 ± 0.37
V	10.22 ± 0.51*	12.09 ± 0.99	40 ± 11
Cr	29.7 ± 5.2*	7.95 ± 0.73	326 ± 11
Mn	102.0 ± 6.5	2.80 ± 0.21	168 ± 101
Fe	109.6 ± 3.4	952 ± 72	3728 ± 1598
Co	27.1 ± 2.4*	0.574 ± 0.070	0.69 ± 0.31
Ni	525 ± 30*	2.70 ± 0.48	8.1 ± 1.7
Cu	25.2 ± 1.2	2.75 ± 0.21	6.38 ± 0.67

**Zn**	120.4 ± 6.3	3.32 ± 0.89	63 ± 20
**Ga**	4.56 ± 0.15*	3.77 ± 0.45	2.78 ± 0.41
**As**	10.46 ± 0.66*	60.6 ± 6.8*	1.74 ± 0.28
**Se**	36.4 ± 8.8*	17.19 ± 0.80*	0.148 ± 0.022
**Rb**	8.84 ± 0.67	0.572 ± 0.055	94 ± 16
**Sr**	4.24 ± 0.20	2.025 ± 0.040	124 ± 10
**Y**	3.034 ± 0.072*	0.372 ± 0.076	26.6 ± 3.4
**Ag**	0.82 ± 0.35*	n.d.	0.176 ± 0.012
**Cd**	15.6 ± 1.7*	2.13 ± 0.21*	1.89 ± 0.39
**In**	n.d.	5.52 ± 0.44*	n.d.
**Cs**	4.60 ± 0.48*	0.0574 ± 0.0016	0.296 ± 0.025
**Ba**	6.66 ± 0.81	3.38 ± 0.15	5.31 ± 0.66
**La**	2.89 ± 0.31*	1.582 ± 0.071	13.9 ± 1.7
**Ce**	9.1 ± 1.0*	4.692 ± 0.085	4.7 ± 1.0
**Pr**	1.05 ± 0.15*	0.4221 ± 0.0086	0.81 ± 0.15
**Nd**	1.54 ± 0.21*	1.499 ± 0.016	2.94 ± 0.55
**Sm**	0.323 ± 0.042*	347.8 ± 3.4*	0.64 ± 0.12
**Eu**	0.320 ± 0.031*	74.24 ± 0.46*	0.432 ± 0.059

**Gd**	0.260 ± 0.039*	254.1 ± 9.0*	0.78 ± 0.14
**Tb**	0.073 ± 0.013*	28.1 ± 2.0*	0.106 ± 0.020
**Dy**	0.198 ± 0.033*	152 ± 13*	0.86 ± 0.16
**Ho**	0.084 ± 0.017*	14.8 ± 1.9*	0.1629 ± 0.032
**Er**	0.099 ± 0.011*	36.2 ± 5.3*	0.53 ± 0.10
**Tm**	0.0293 ± 0.0045*	2.63 ± 0.85*	72 ± 15*
**Yb**	0.084 ± 0.012	0.0292 ± 0.0033	1.21 ± 0.20
**Lu**	0.0264 ± 0.0045*	1.63 ± 0.51*	75 ± 18*
**Tl**	0.390 ± 0.069*	n.d.	0.054 ± 0.031
**Pb**	8.1 ± 5.6*	828 ± 32*	0.296 ± 0.087
**Bi**	0.18 ± 0.12*	21.32 ± 0.34	n.d.
**Th**	n.d.	1.611 ± 0.095	0.43 ± 0.11
**U**	1.11 ± 0.31*	0.275 ± 0.011	14.7 ± 4.6

* Values expressed in ng/g.

Table S2. Elements accumulated in adults of *Tenebrio molitor* treated with metribuzin-based commercial formulation. Mean concentrations and standard deviation of the analyzed elements for each group of beetles (20 samples for each group, results on a dry-weight basis). N.d.: not detected. [FC, female control; FT, female treated; MC, male control; MT, male treated; 2d, two days; 7d, seven days]. n.d.: not detected

	**FC_2d**	**FC_7d**	**FT_2d**	**FT_7d**	**MC_2d**	**MC_7d**	**MT_2d**	**MT_7d**
**Na [µg/g]**	1686 ± 207	1730 ± 82	2038 ± 240^{ac}	1924 ± 241	1832 ± 289	1972 ± 194	1604 ± 223 ^{abc}	1975 ± 275^{b}
**Mg [µg/g]**	2106 ± 426	2793 ± 629	2543 ± 406 ^{abc}	2116 ± 244 ^{abc}	1026 ± 127	1122 ± 167	1247 ± 429 ^{c}	1155 ± 170 ^{c}
**Al [µg/g]**	3.6 ± 1.2	5.1 ± 1.5	4.4 ± 1.9	4.1 ± 1.5 ^{c}	3.3 ± 1.4	1.2 ± 1.1	3.2 ± 1.9 ^{b}	1.15± 0.49 ^{bc}
**K [µg/g]**	10069 ± 1333	10885 ± 525	11481 ± 1319 ^{ac}	11270 ± 949 ^{c}	6009 ± 555	6279 ± 692	5127 ± 732 ^{abc}	6228 ± 646 ^{bc}
**Ca [µg/g]**	59 ± 11	58.6 ± 8.4	68 ± 12 ^{abc}	52.0 ± 5.9 ^{b}	47.0 ± 7.2	40.3 ± 4.4	42.3 ± 6.8 ^{c}	46.6 ± 8.5
**Mn [µg/g]**	9.9 ± 1.3	11.7 ± 1.3	11.3 ± 2.1 ^{c}	11.11 ± 0.89	9.9 ± 1.2	11.9 ± 1.1	8.8 ± 2.0 ^{bc}	11.2 ± 1.5 ^{b}
**Fe [µg/g]**	67.1 ± 9.5	77.3 ± 8.1	76 ± 11 ^{c}	72.1 ± 9.9	58.4 ± 9.0	71 ± 13	54.7 ± 6.0 ^{bc}	66.0 ± 8.4 ^{b}
**Cu [µg/g]**	18.8 ± 3.5	24.6 ± 2.4	20.6 ± 2.5 ^{c}	22.4 ± 2.4 ^{c}	34.7 ± 6.0	47.8 ± 6.5	33.5 ± 4.1 ^{bc}	45.7 ± 4.2 ^{bc}
**Zn [µg/g]**	99 ± 11	103.8 ± 5.7	105.3 ± 8.0 ^{c}	109 ± 13 ^{c}	206 ± 24	192 ± 26	191± 14 ^{bc}	224 ± 21 ^{abc}
**Be [pg/g]**	n.d.	n.d.	n.d.	n.d.	0.64 ± 1.65	0.29 ± 0.94	n.d.	0.6 ± 1.3
**V [pg/g]**	5.2 ± 2.3	6.5 ± 2.0	6.0 ± 2.7 ^{c}	5.4 ± 1.0 ^{c}	3.28 ± 0.91	2.1 ± 1.3	1.43 ± 0.93 ^{ac}	0.23 ± 0.18 ^{ac}
**Cr [pg/g]**	196 ± 687	160 ± 93	22 ± 18	51 ± 23	8 ± 11	87 ± 46	77 ± 137	n.d.

**Co [pg/g]**	7.0 ± 2.3	13.6 ± 2.5	10.0 ± 4.4	12.7 ± 2.7 [c]	7.5 ± 2.6	11.5 ± 1.8	5.9 ± 1.0 [b]	24 ± 14 [a b c]
**Ni [pg/g]**	81 ± 171	614 ± 301	50 ± 48 [b]	333 ± 69 [a b]	127 ± 80	205 ± 41	55 ± 50 [b]	259 ± 140 [b]
**Ga [pg/g]**	2.04 ± 0.49	4.11 ± 0.52	3.9 ± 1.1 [a c]	3.50 ± 0.51 [a c]	1.09 ± 0.30	0.90 ± 0.22	0.76 ± 0.26 [c]	0.28 ± 0.32 [a c]
**As [pg/g]**	1.7 ± 1.9	0.91 ± 0.78	n.d.	n.d.	3.78 ± 0.78	4.22 ± 0.69	2.88 ± 0.82 [c]	3.31 ± 0.86 [a c]
**Se [pg/g]**	178 ± 40	149 ± 15	115 ± 35 [a]	128± 35 [c]	74 ± 18	61.1 ± 8.2	91 ± 11	71 ± 26 [c]
**Rb [pg/g]**	302 ± 160	786 ± 128	348 ± 256 [b]	1098 ± 349 [a b c]	432 ± 182	1171 ± 250	348 ± 199 [b]	1123 ± 274 [b c]
**Sr [pg/g]**	1293 ± 477	1174 ± 212	1428 ± 675 [c]	1247 ± 316 [c]	932 ± 393	1097 ± 212	893 ± 222 [c]	809 ± 192 [c]
**Y [pg/g]**	1.62 ± 0.53	1.54 ± 0.43	1.70 ± 0.56 [c]	1.11 ± 0.23 [c]	1.25 ± 0.34	0.39 ± 0.25	5.2 ± 6.5 [b c]	0.55 ± 0.16 [b c]
**Ag [pg/g]**	n.d.	n.d.	n.d.	n.d.	1.0 ± 3.4	1.17 ± 0.59	n.d.	n.d.
**Cd [pg/g]**	29 ± 10	28.5 ± 5.2	30.0 ± 9.8	23.4 ± 6.0 [c]	33 ± 14	33 ± 14	19.4 ± 6.0 [a b]	36 ± 19 [b c]
**Cs [pg/g]**	3.2 ± 2.7	n.d.	0.07 ± 0.23 [a]	0.10 ± 0.24	0.39 ± 0.19	0.43 ± 0.24	0.06 ± 0.11	0.32 ± 0.29
**Ba [pg/g]**	194 ± 90	654 ± 135	279 ± 238 [b c]	532 ± 119 [b]	113 ± 47	415 ± 105	97± 42 [b c]	609 ± 396 [a b]
**La [pg/g]**	1.65 ± 0.69	3.9 ± 2.0	1.3 ± 0.69	1.00 ± 0.28 [a]	1.8 ± 1.0	0.41 ± 0.45	0.71 ± 0.29 [a]	0.78 ± 0.58
**Ce [pg/g]**	4.1 ± 1.5	4.3 ± 2.2	3.6 ± 1.7	2.67 ± 0.78	5.6 ± 3.3	1.5 ± 1.4	2.22 ± 0.78 [a]	1.8 ± 1.1
**Pr [pg/g]**	0.36 ± 0.17	0.32 ± 0.20	0.32 ± 0.21	0.29 ±0.18	0.38 ± 0.13	0.081 ± 0.077	0.244 ± 0.086	0.23 ± 0.10
**Nd [pg/g]**	1.54 ± 0.63	1.13 ± 0.40	1.42 ± 0.70 [c]	1.08 ± 0.51 [c]	0.63 ± 0.17	0.16 ± 0.12	0.42 ± 0.15 [c]	0.29 ± 0.12 [c]

**Pm [pg/g]**	1.31 ± 0.62	1.04 ± 0.41	1.09 ± 0.69 [c]	1.01 ± 0.52	0.74 ± 0.29	0.08 ± 0.14	0.52 ± 0.21 [c]	0.67 ± 0.26 [a]
**Sm [pg/g]**	0.26 ± 0.14	0.101 ± 0.080	0.19 ± 0.15 [b c]	0.10 ± 0.10 [b]	0.119 ± 0.049	0.020± 0.023	0.059 ± 0.026 [c]	0.032 ± 0.028
**Eu [pg/g]**	0.004 ± 0.013	0.042 ± 0.028	0.010 ± 0.032 [c]	0.018 ± 0.016	n.d.	n.d.	0.16 ± 0.29 [a b c]	n.d.
**Gd [pg/g]**	0.17 ± 0.12	0.058 ± 0.046	0.14 ± 0.14 [b c]	0.055 ± 0.062 [b]	0.091 ± 0.029	0.034 ± 0.025	0.055 ± 0.025 [c]	0.037 ± 0.024
**Tb [pg/g]**	n.d.	n.d.	n.d.	n.d.	0.025 ± 0.017	n.d.	0.013 ± 0.015 [c]	0.005 ± 0.014
**Dy [pg/g]**	0.26 ± 0.61	0.080 ± 0.044	0.085 ± 0.090	0.064 ± 0.035	0.100 ± 0.036	0.031 ± 0.013	0.062 ± 0.033	0.042 ± 0.028
**Ho [pg/g]**	n.d.	n.d.	n.d.	n.d.	0.034 ± 0.019	0.019 ± 0.018	0.018 ± 0.014 [a b c]	n.d.
**Er [pg/g]**	0.023 ± 0.038	0.0005 ± 0.0010	0.014 ± 0.028	0.0036 ± 0.0089 [c]	0.051 ± 0.022	0.023 ± 0.019	0.021 ± 0.017 [a]	0.025 ± 0.020 [c]
**Tm [pg/g]**	n.d.	n.d.	n.d.	n.d.	0.011 ± 0.011	n.d.	0.006 ± 0.011 [b c]	n.d.
**Yb [pg/g]**	0.004 ± 0.014	n.d.	0.0003 ± 0.0016 [c]	n.d.	0.050 ± 0.028	0.025 ± 0.021	0.022 ± 0.011 [a b c]	n.d.
**Hg [pg/g]**	354 ± 513	4954 ± 1915	565 ± 780 [c]	2218 ± 923 [c]	1300 ± 1271	n.d.	6197 ± 6610 [a c]	7807 ± 5255 [a c]
**Tl [pg/g]**	0.029 ± 0.131	n.d.	n.d.	n.d.	0.173 ± 0.068	n.d.	0.134 ± 0.071 [b c]	0.039 ± 0.029
**Pb [pg/g]**	5.5 ± 9.6	12.4 ± 7.5	0.4 ± 1.6 [b]	24.4 ± 8.0 [a b]	13 ± 13	20 ± 16	9.3 ± 7.8 [b]	23 ± 12
**Bi [pg/g]**	n.d.	n.d.	n.d.	n.d.	0.91 ± 0.56	3.9 ± 3.8	1.7 ± 2.5 [c]	0.67 ± 0.53 [a]
**U [pg/g]**	0.07 ± 0.14	0.053 ± 0.055	0.013 ± 0.040 [c]	0.065 ± 0.058 [c]	0.48 ± 0.33	0.54 ± 0.26	0.24 ± 0.17 [a c]	0.30 ± 0.28 [a c]

[a] significantly different values from the corresponding control at the same time point (Tukey test $p<0.05$)

[b] significantly different values within the same sex at 2 and 7 days (Tukey test $p<0.05$).

[c] significantly different values between treated males and females at the same time point (Tukey test $p<0.05$).

Table S3. Elements accumulated in adults of *Tenebrio molitor* treated with NPK fertilizer. Mean concentrations and standard deviation of the analyzed elements for each group of beetles (20 samples for each group, results on a dry-weight basis). N.d.: not detected. [FC: female control; FT: female treated; MC: male control; MT: male treated; 2d: two days; 7d: seven days]. n.d.: not detected

	**FC_2d**	**FC_7d**	**FT_2d**	**FT_7d**	**MC_2d**	**MC_7d**	**MT_2d**	**MT_7d**
**Na [µg/g]**	1758 ± 262	1852 ± 379	2045 ± 342^{ab}	1758 ± 227^{bc}	2215 ± 236	2147 ± 299	1947 ± 315	2047 ± 261^{c}
**Mg [µg/g]**	960 ± 162	1009 ± 184	1384 ± 407^{abc}	1143 ± 318^{b}	1277 ± 181	1110 ± 79	997 ± 179^{ac}	1124 ± 323
**Al [µg/g]**	1.47 ± 0.90	n.d.	1.38 ± 0.76^{b}	3.9 ± 1.6^{abc}	1.19 ± 0.54	1.19 ± 0.40	0.97 ± 0.51^{b}	3.0 ± 1.4^{abc}
**K [µg/g]**	4433 ± 459	4748 ± 526	5288 ± 706^{a}	4754 ± 447^{c}	6277 ± 480	5262 ± 491	5832 ± 659	5691 ± 774^{c}
**Ca [µg/g]**	42.5 ± 6.1	43.1 ± 7.9	52.0 ± 7.4	56 ± 12^{a}	64 ± 14	47.4 ± 7.2	53.2 ± 9.2^{a}	62 ± 16^{a}
**Mn [µg/g]**	8.5 ± 1.3	10.2 ± 2.2	11.7 ± 3.6^{ac}	10.1 ± 2.1	10.3 ± 1.9	13.3 ± 3.0	8.9 ± 1.7^{c}	9.8 ± 1.8^{a}
**Fe [µg/g]**	63 ± 11	65 ± 10	56.4 ± 9.5b	69 ± 11b	59 ± 10	67 ± 13	59 ± 11^{b}	71.3 ± 9.3^{b}
**Cu [µg/g]**	36.0 ± 4.3	38.5 ± 3.8	32.2 ± 3.5	35.9 ± 6.1	41.1 ± 6.1	42.5 ± 5.9	35.6 ± 5.4^{a}	37.7 ± 5.7
**Zn [µg/g]**	180 ± 14	192 ± 18	171 ± 16^{bc}	194 ± 25b	197 ± 17	202 ± 18	198 ± 30^{c}	197 ± 23
**Be [ng/g]**	n.d.	n.d.	1.0 ± 1.1^{abc}	n.d.	n.d.	n.d.	2.4 ± 1.9^{abc}	0.25 ± 0.77^{b}
**V [ng/g]**	n.d.	1.56 ± 0.64	18 ± 17^{b}	111 ± 68^{ab}	0.62 ± 0.41	2.29 ± 0.80	11.1 ± 6.5b	124 ± 86^{ab}
**Cr [ng/g]**	29 ± 25	157 ± 168	5.5 ± 13.6^{b}	820 ± 449^{ab}	6.1 ± 8.3	25 ± 22	161 ± 89b	829 ± 646^{ab}
**Co [ng/g]**	3.2 ± 3.4	11.6 ± 1.7	12.9 ± 4.3^{ac}	12.5 ± 4.5^{c}	14.4 ± 7.0	11.9 ± 1.9	19.1 ± 8.1^{c}	17.8 ± 4.7^{ac}
**Ni [ng/g]**	142 ± 450	168 ± 37	63 ± 49	197 ± 69	16 ± 14	171 ± 43	34 ± 20	190 ± 97
**Ga [ng/g]**	n.d.	0.049 ± 0.219	2.9 ± 2.2^{ab}	5.6 ± 4.5^{abc}	0.20 ± 0.28	0.61 ± 0.24	1.4 ± 0.7^{b}	9.8 ± 6.1^{abc}
**As [ng/g]**	1.0 ± 1.4	3.32 ± 0.61	8.8 ± 1.5^{ab}	17 ± 11^{ab}	6.0 ± 1.4	3.37 ± 0.70	8.4 ± 3.0^{b}	19 ± 14^{ab}
**Se [ng/g]**	111 ± 28	92 ± 14	101 ± 22	88 ± 15	62 ± 17	90 ± 11	92 ± 13^{a}	97 ± 23
**Rb [ng/g]**	512 ± 247	585 ± 115	1022 ± 689^{ac}	817 ± 303	941 ± 696	975 ± 185	469 ± 177^{ac}	808 ± 437
**Sr [ng/g]**	1184 ± 351	940 ± 259	1336 ± 537^{c}	1120 ± 399	927 ± 183	1202 ± 289	888 ± 281^{c}	1076 ± 448
**Y [ng/g]**	n.d.	1.3 ± 1.4	30 ± 23^{b}	97 ± 70^{ab}	0.98 ± 0.77	0.68 ± 0.28	16 ± 10^{b}	84 ± 67^{ab}
**Ag [ng/g]**	n.d.	n.d.	n.d.	n.d.	n.d.	0.97 ± 0.45	1.9 ± 1.1^{abc}	4.4 ± 2.9^{abc}
**Cd [ng/g]**	18 ± 13	24 ± 10	24 ± 10	45 ± 14^{a}	39 ± 17	26.1 ± 9.5	37 ± 14^{a}	49 ± 18^{a}

**In [ng/g]**	n.d.	0.0021 ± 0.0092	0.044 ± 0.034[c]	n.d.	n.d.	0.19 ± 0.17	0.18 ± 0.16[abc]	n.d.
**Cs [ng/g]**	0.25 ± 1.12	0.03 ± 0.15	0.93 ± 0.69	0.62 ± 0.80[c]	0.32 ± 0.43	0.49 ± 0.14	0.42 ± 0.68[b]	2.6 ± 1.5[abc]
**Ba [ng/g]**	217 ± 112	465 ± 156	246 ± 149	239 ± 58[a]	182 ± 103	509 ± 177	131 ± 70[b]	311 ± 157[ab]
**La [ng/g]**	n.d.	0.37 ± 0.27	8.0 ± 6.7[b]	20 ± 16[abc]	0.42 ± 0.21	0.43 ± 0.21	5.1 ± 3.0[b]	31 ± 20[abc]
**Ce [ng/g]**	n.d.	1.6 ± 1.2	11.2 ± 8.8[b]	41 ± 26[ab]	1.02 ± 0.55	1.36 ± 0.51	6.7 ± 3.9[b]	34 ± 25[ab]
**Pr[ng/g]**	n.d.	0.068 ± 0.098	2.4 ± 1.9[b]	5.3 ± 4.8[ab]	0.10 ± 0.05	0.14 ± 0.05	1.41 ± 0.81[b]	7.6 ± 5.7[ab]
**Nd [ng/g]**	n.d.	0.15 ± 0.18	4.5 ± 3.6[b]	17.4 ± 9.8[ab]	0.26 ± 0.14	0.255 ± 0.079	2.7 ± 1.2[b]	13.3 ± 9.1[ab]
**Pm [ng/g]**	n.d.	0.14 ± 0.26	6.4 ± 5.1[b]	18 ± 12[ab]	0.33 ± 0.17	0.44 ± 0.16	3.6 ± 2.1[b]	22 ± 13[ab]
**Sm [ng/g]**	n.d.	0.0022 ± 0.0102	0.97 ± 0.91[b]	2.7 ± 2.1[ab]	0.040 ± 0.026	0.038 ± 0.017	0.57 ± 0.35[b]	3.3 ± 2.4[ab]
**Eu [ng/g]**	n.d.	n.d.	0.24 ± 0.22	n.d.	0.0046 ± 0.0209	n.d.	0.27 ± 0.15[b]	1.14 ± 0.79[abc]
**Gd [ng/g]**	n.d.	n.d.	1.4 ± 1.1[b]	4.2 ± 3.0[ab]	0.04 ± 0.02	0.02 ± 0.01	0.68 ± 0.41[b]	3.7 ± 2.7[ab]
**Tb [ng/g]**	n.d.	n.d.	0.47 ± 0.36[a]	0.36 ± 0.67[c]	0.013 ± 0.015	0.0013 ± 0.0061	0.23 ± 0.13[b]	1.6 ± 0.9[abc]
**Dy [ng/g]**	n.d.	0.023 ± 0.033	1.3 ± 1.0[b]	3.7 ± 2.3[ab]	0.024 ± 0.008	0.023 ± 0.010	0.71 ± 0.44[b]	4.2 ± 2.9[ab]
**Ho [ng/g]**	n.d.	n.d.	0.57 ± 0.48[b]	1.6 ± 1.4[ab]	n.d.	0.021 ± 0.019	0.38 ± 0.19[b]	2.0 ± 1.4[ab]
**Er [ng/g]**	n.d.	0.0020 ± 0.0089	0.98 ± 0.82[b]	2.7 ± 2.1[ab]	0.0061 ± 0.0097	0.012 ± 0.012	0.62 ± 0.35[b]	3.0 ± 2.1[ab]
**Tm [ng/g]**	n.d.	0.003 ± 0.013	0.25 ± 0.20	0.18 ± 0.37	0.0062 ± 0.0098	0.0012 ± 0.0053	0.15 ± 0.10[b]	0.89 ± 0.68[ab]
**Yb [ng/g]**	n.d.	n.d.	1.04 ± 0.81	1.0 ± 1.7[c]	0.0008 ± 0.0035	n.d.	0.64 ± 0.34[b]	3.5 ± 2.4[abc]
**Lu [ng/g]**	n.d.	n.d.	0.34 ± 0.25[a]	0.24 ± 0.48[c]	n.d.	n.d.	0.18 ± 0.10[b]	1.26 ± 0.84[abc]
**Hg [ng/g]**	32 ± 145	26 ± 119	376 ± 374b	1064 ± 1021[abc]	n.d.	83 ± 210	745 ± 412[a]	548 ± 655[ac]
**Tl [ng/g]**	n.d.	n.d.	0.57 ± 0.33[a]	0.048 ± 0.217[c]	0.42 ± 0.30	0.21 ± 0.12	0.79 ± 0.47[ab]	2.2 ± 1.3[abc]
**Pb [ng/g]**	0.05 ± 0.22	2.2 ± 1.4	n.d.	5.4 ± 2.7[b]	1.6 ± 2.2	2.4 ± 2.8	9.4 ± 10.6[abc]	5.0 ± 2.5[b]
**Bi [ng/g]**	n.d.	0.080 ± 0.206	0.22 ± 0.13[c]	n.d.	0.25 ± 0.26	0.70 ± 0.71	0.86 ± 0.55[abc]	0.19 ± 0.18[ab]
**Th [ng/g]**	n.d.	n.d.	n.d.	n.d.	n.d.	n.d.	0.7 ± 1.5[abc]	n.d.
**U [ng/g]**	n.d.	0.09 ± 0.18	22 ± 19[b]	98 ± 67[ab]	0.045 ± 0.056	2.7 ± 2.7	13.0 ± 7.9[b]	116 ± 74[ab]

[a] significantly different values from the corresponding control at the same time point (Tukey test p<0.05)

[b] significantly different values within the same sex at 2 and 7 days (Tukey test p<0.05).

[c] significantly different values between treated males and females at the same time point (Tukey test p<0.05).

Table S4. Principal axis factoring for metribuzin-based herbicide treated adults of *Tenebrio molitor*, matrix with factor loadings. Loadings below 0.5 were not considered to improve the understanding of the data structure.

	**FACTOR MATRIX**									
	1	2	3	4	5	6	7	8	9	10
**Pm**	.858									
**Nd**	.842									
**Sm**	.812									
**Pr**	.794									
**Gd**	.782									
**V**	.684									
**Al**	.669									
**Ce**	.665									
**La**	.513									.513
**Dy**										
**K**		.686								
**Ca**		.677								
**Sr**		.654								
**Mg**		.642								
**Zn**		-.631								
**Ga**		.603								
**As**		-.573								

**Cu**	-.540							
**Se**								
**Be**								
**Ho**		.673	.580					
**Yb**		.664						
**Bi**		.651						
**U**		.591						
**Ag**								
**Tb**			.749					
**Tm**			.728					
**Tl**			.598					
**Er**								
**Rb**				.722				
**Ba**				.707				
**Pb**				.662				
**Co**								
**Eu**					.909			
**Y**					.886			
**Hg**								
**Mn**						.602		
**Na**								
**Cd**								
**Fe**								
**Ni**				.510			.595	
**Cr**								
**Cs**								.732

Table S5 Principal axis factoring for NPK fertilize treated adults of *Tenebrio molitor*, matrix with factor loadings. Loadings below 0.5 were not considered to improve the understanding of the data structure.

	FACTOR MATRIX							
	1	2	3	4	5	6	7	8
**Nd**	1.049							
**Al**	1.035							
**Ce**	1.018							
**Gd**	.962							
**Dy**	.895							
**Y**	.865							
**V**	.849							
**Ho**	.838							
**Sm**	.832							
**Cr**	.814							
**U**	.805							
**Pm**	.805							
**Er**	.794							
**As**	.671							
**La**	.656							
**Hg**	.639							
**Ga**	.634							
**Pr**	.578							
**Tl**		.882						
**Eu**		.875						

**Tb**	.797					
**Lu**	.769					
**Ag**	.719					
**Cs**	.695					
**Tm**	.677					
**Yb**	.580					
**In**		.826				
**Be**		.630				
**Pb**		.585				
**Bi**		.576				
**Th**						
**Mg**			.712			
**K**			.599			
**Na**			.540			
**Rb**						
**Ca**						
**Sr**						
**Ba**				.758		
**Mn**				.557		
**Ni**						
**Fe**						
**Zn**					.767	
**Cu**						
**Co**						.563
**Cd**						.546
**Se**						-.537

.
